# TRPC3 and TRPC6 are essential for normal mechanotransduction in subsets of sensory neurons and cochlear hair cells

**DOI:** 10.1098/rsob.120068

**Published:** 2012-05

**Authors:** Kathryn Quick, Jing Zhao, Niels Eijkelkamp, John E. Linley, Francois Rugiero, James J. Cox, Ramin Raouf, Martine Gringhuis, Jane E. Sexton, Joel Abramowitz, Ruth Taylor, Andy Forge, Jonathan Ashmore, Nerissa Kirkwood, Corné J. Kros, Guy P. Richardson, Marc Freichel, Veit Flockerzi, Lutz Birnbaumer, John N. Wood

**Affiliations:** 1 Molecular Nociception Group, Wolfson Institute for Biomedical Research, University College London, London WC1E 6BT, UK; 2 Laboratory of Neurobiology, National Institute of Environmental Health Sciences, NIH, Building 101, Room F180, 111 TW Alexander Dr, Research Triangle Park, NC 27709, USA; 3 UCL Ear Institute, 332 Gray's Inn Road, London WC1X 8EE, UK; 4 School of Life Sciences, University of Sussex, Brighton BN1 9QG, UK; 5 Department of Experimental and Clinical Pharmacology and Toxicology, Faculty of Medicine, Saarland University, Homburg, Germany; 6 WCU Programme, Department of Molecular Medicine, Seoul National University, Seoul 151-742, South Korea

**Keywords:** mechanosensation, touch, hearing

## Abstract

Transient receptor potential (TRP) channels TRPC3 and TRPC6 are expressed in both sensory neurons and cochlear hair cells. Deletion of TRPC3 or TRPC6 in mice caused no behavioural phenotype, although loss of TRPC3 caused a shift of rapidly adapting (RA) mechanosensitive currents to intermediate-adapting currents in dorsal root ganglion sensory neurons. Deletion of both TRPC3 and TRPC6 caused deficits in light touch and silenced half of small-diameter sensory neurons expressing mechanically activated RA currents. Double TRPC3/TRPC6 knock-out mice also showed hearing impairment, vestibular deficits and defective auditory brain stem responses to high-frequency sounds. Basal, but not apical, cochlear outer hair cells lost more than 75 per cent of their responses to mechanical stimulation. FM1-43-sensitive mechanically gated currents were induced when TRPC3 and TRPC6 were co-expressed in sensory neuron cell lines. TRPC3 and TRPC6 are thus required for the normal function of cells involved in touch and hearing, and are potential components of mechanotransducing complexes.

## 2. Introduction

The identification of mammalian ion channels that transduce mechanical stimuli involved in touch and hearing has proved problematic. Many ion channels appear to be mechanically gated. Two pore potassium channels and the large hydrophobic proteins Piezo1 and 2 confer mechanosensitivity on cells in which they are expressed, and several members of the transient receptor potential (TRP) channel family have been implicated in mechanosensation [1–4]. Ion channels linked to deafness include a number of potassium channels and both TRPML3 and TRPV4, although none of these channels are a strong candidate for primary mechanotransduction, because of their localization or biophysical properties [5]. Recent work on TMC1 and TMC2 has shown a requirement for these transmembrane channel-like proteins in cochlear mechanotransduction where they are expressed at the tip links of cochlear hair cells [6]. However, they have yet to be shown to be mechanosensitive channels in heterologous expression systems.

In worms and flies, probable mechanotransducers have been identified using genetic screens. Since the cloning of the first TRP channel involved in *Drosophila* vision, a number of invertebrate TRP channels have been implicated in mechanotransduction. NOMPC (no mechanoreceptor potential C) mutants in *Drosophila* show deficits in mechanosensation, while the painless TRPA mutant loses responses to noxious mechanical pressure and heat. Two members of the TRPV family, Nanchung and Inactive, are found in *Drosophila* chordotonal organs and are required for hearing in flies [7–9]. In the nematode worm *Caenorhabditis elegans*, an analysis of mechanosensitive mutants has led to the identification of transducing Mec channels that are members of the epithelial sodium channel (ENaC) family. However, mammalian ENaC family members do not seem to be mechanosensors in the somatosensory system [1,10]. The TRP family members OSM-9 and TRPA1 have been implicated in osmosensation and touch sensation [1], while the strongest evidence of a role for a TRP channel as a direct mechanotransducer comes from mutagenesis studies of the *C. elegans* TRP-4 channel, a member of the TRPN set that is not expressed in mammals [11].

TRP channels are thus candidate mammalian mechanosensors. TRPV4 deletion in mice is associated with defective responses to noxious mechanical pressure and late-onset deafness [12]. TRPA1, a mammalian channel activated by environmental irritants, has structural features reminiscent of invertebrate mechanosensors, and deletion of the gene causes deficits in mechanosensation in mice [13,14]. Although hearing is apparently normal in the absence of TRPA1 [13], noxious mechanosensation is blunted and neurons that express slowly adapting (SA) mechanosensitive currents are silenced [14]. In addition, TRPA1 blockers inhibit mechanical hyperalgesia [15]. Members of the broadly expressed canonical TRP (TRPC) family of cation channels have also been shown to be activated by mechanical stimuli [16]. In the cardiovascular system, indirect activation of TRPC6 through G_q/11_ protein activation via the Angiotensin II (AT1) receptor has been described, and this slowly developing current is induced by mechanical pressure in the absence of a ligand. It has been proposed that membrane deformation induces the same structural alterations as ligand binding to the AT1 receptor [17–19]. TRPC1 has been implicated in low-threshold mechanosensation in dorsal root ganglion (DRG) neurons [20]. TRPC6 has also been found in mechanosensory complexes in the kidney. The membrane protein Podocin associates with and regulates TRPC6 as part of a complex also including Neph1, Neph2, Nephrin and CD2AP, which may act to sense glomerular pressure [21]. In contrast, there is no evidence for or against a role for TRPC3 as a mechanosensor.

We and others have found selective expression of TRPC3 and TRPC6 in small-diameter sensory neurons of mouse dorsal root ganglia [22,23]. TRPC3 and TRPC6 are known to heteromultimerize, and can be co-immunoprecipated from rat brain [24,25]. Because there is evidence that these channels show some functional redundancy, we examined single and double knock-out (DKO) animals for deficits in somatosensation and pain behaviour [16]. We found that single null mutants had no behavioural deficits, but double mutants showed a partial but consistent loss of sensitivity to the application of innocuous mechanical pressure. In contrast, acute heat-sensing and responses to noxious mechanical pressure were normal. We noticed that the double but not single knock-out animals also appeared to have a hearing loss. Here, we present evidence that a loss of mechanotransduction contributes to the touch and hearing deficits apparent in TRPC3/TRPC6 DKO mice.

## 3. Results

### 3.1. TRPC3 and TRPC6 are co-expressed in adult dorsal root ganglion sensory neurons

**Figure 1.**
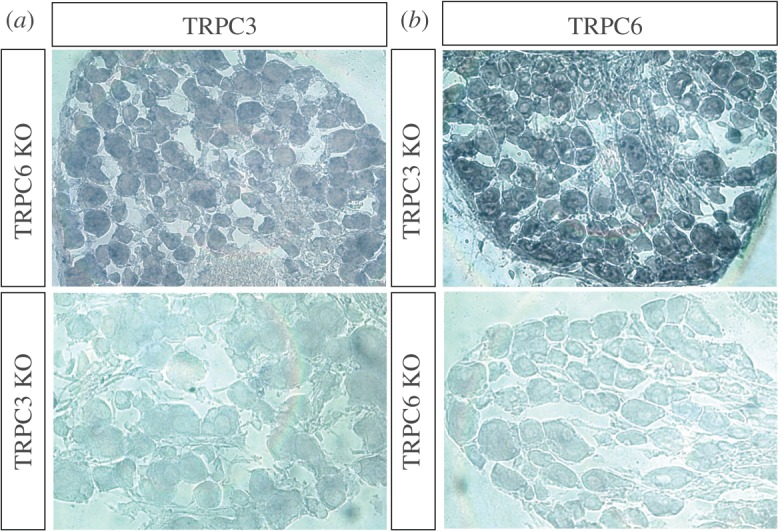
TRPC3 and TRPC6 are expressed in DRG neurons. *In situ* hybridization shows that TRPC3 (*a*) and TRPC6 (*b*) transcripts are found in almost all adult DRG neurons. The antisense TRPC3 probe does not stain neurons from TRPC3 knock-out mice and the antisense TRPC6 probe does not stain neurons from TRPC6 knock-out mice. See also electronic supplementary material, figure S1.

Microarray studies of Na_v_1.8+ neurons and *in situ* hybridization studies of DRG neurons have shown that they express high levels of both TRPC3 and TRPC6 transcripts [22]. Immunohistochemical studies of TRP channels have been compromised by the lack of availability of specific antibodies. When we checked commercially available antisera to TRPC3 and TRPC6, they all stained knock-out tissue (not shown). We therefore used *in situ* hybridization with cRNA probes to TRPC3 and TRPC6, and found that both transcripts were present in all sensory neurons within DRG from 12-week-old mice, and the signals were lost in tissue from knock-out mice (figure 1). All postnatal DRG neurons were positive for TRPC3 transcripts in another study, but expression declined with age [23]. The *in situ* probes were transcript-specific, as shown by an analysis of expression in single knock-out mice.

We also examined the possible effect of channel deletion on sensory neuron numbers. There was no loss in cell numbers in the dorsal root ganglia of TRPC3/TRPC6 DKO mice (see electronic supplementary material, figure S1*a*). The relative percentage of sensory neuron subsets defined with markers for neurofilaments, peripherin, calcitonin gene-related peptide (CGRP) or isolectin B4 (IB4) binding were the same in wild-type (WT) and DKO mice. WT control and TRPC3/TRPC6 DKO mice had a similar percentage of peripherin, neurofilament 200 and peripherin + neurofilament 200 positive DRG neurons (see electronic supplementary material, figure S1*b*,*c*). The IB4 and CGRP staining show that terminals of sensory neurons in the dorsal horn were also normal (see electronic supplementary material, figure S1*d*). Thus deletion of TRPC3 and TRPC6 does not appear to compromise sensory neuron viability.

### 3.2. Adult TRPC3/TRPC6 double knock-outs show selective deficits in touch responsiveness

**Figure 2.**
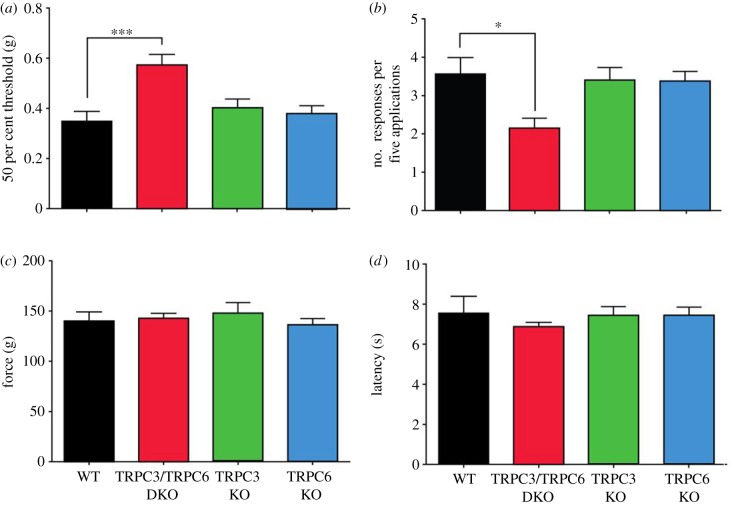
Selective deficits in innocuous mechanosensation in TRPC3/TRPC6 double knock-out (DKO) mice. (*a*) Fifty per cent threshold to mechanical stimulation using von Frey hairs in wild-type (WT) (*n* = 8), TRPC3/TRPC6 DKO (*n* = 9), TRPC3 knock-out (KO) (*n* = 7) and TRPC6 KO (*n* = 10) mice. (*b*) Responses of WT, TRPC3/TRPC6 DKO, TRPC3 KO and TRPC6 KO mice to a cotton bud applied to the plantar surface of the hind paw (*n* = 6/group). (*c*) Response to noxious mechanical stimulation using a Randall–Selitto apparatus in WT (*n* = 7), TRPC3/TRPC6 DKO (*n* = 9), TRPC3 KO (*n* = 6) and TRPC6 KO (*n* = 7) mice. (*d*) Response to noxious thermal stimulation using Hargreaves’ apparatus in TRPC3/TRPC6 DKO (*n* = 14), TRPC3 KO (*n* = 11), TRPC6 KO (*n* = 15) and WT (*n* = 12) mice. Data are expressed as mean ± s.e.m. *p < 0.05; **p < 0.01; ***p < 0.001. See also electronic supplementary material, figure S2.

We examined global TRPC3 and TRPC6 knock-out mice, as well as DKO mice in a range of behavioural assays [22]. Motor co-ordination on a rotarod was normal in all mouse lines (see electronic supplementary material, figure S2), allowing us to test responses to mechanical and thermal stimuli. Withdrawal responses to von Frey hairs or detection of a cotton bud (a recent assay described in Garrison *et al.* [20]) were blunted in double but not single knock-out mice, indicating a deficit in mechanosensation (figure 2*a*,*b*). However, threshold responses to noxious mechanical pressure applied with a Randall–Selitto apparatus (figure 2*c*), as well as threshold responses to noxious heat applied with a Hargreaves apparatus (figure 2*d*) were normal in single and double TRPC3/TRPC6 knock-outs.

### 3.3. Adult TRPC3/TRPC6 double knock-outs show electrophysiological deficits in mechanotransduction

**Figure 3.**
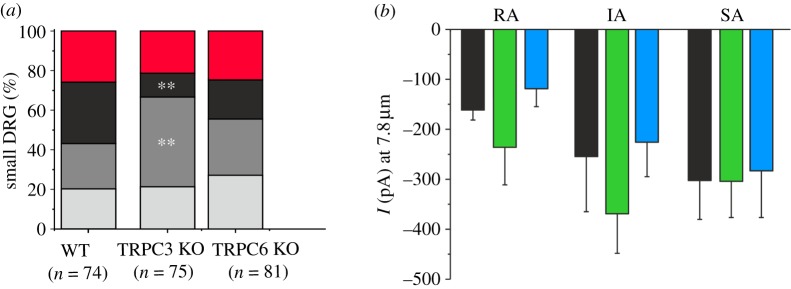
Electrophysiological characterization of sensory neurons of single TRPC3 and TRPC6 knock-out mice. Mechanically evoked currents recorded from small-diameter DRG neurons with broad action potentials using the whole-cell patch clamp technique and classified based on their adaptation kinetic to a static mechanical stimulus applied to the soma. Currents are defined as rapidly adapting (RA), intermediately adapting (IA) and slowly adapting (SA). (*a*) The proportion of neurons expressing each current type from WT mice, TRPC3 knock-out mice and TRPC6 knock-out mice is shown. TRPC6 knock-out mice were statistically indistinguishable from WT (red, no response; black, RA; dark grey, IA; light grey, SA). (*b*) The magnitude of mechanically evoked peak currents in small-diameter DRG neurons voltage clamped at −70 mV. No significant difference between genotypes was observed (ANOVA, *p* > 0.05). Data are expressed as mean ± s.e.m.; **p < 0.01 (black bars, WT; green bars, TRPC3 KO; blue bars, TRPC6 KO).

DRG neurons in culture express three types of non-selective cationic currents on mechanical stimulation [26,27]. Large-diameter neurons with narrow action potentials all express rapidly adapting (RA) mechanosensitive currents [26], while small-diameter neurons with broad action potentials may be mechano-insensitive or express SA, intermediately adapting (IA) or RA currents [26]. Using the perforated patch configuration of the whole-cell recording technique, neurons were mechanically distended with a fire-polished pipette [26]. Single TRPC3 or TRPC6 knock-out mice had equivalent numbers of mechanically non-responsive small-diameter DRG neurons as control mice (figure 3*a*). Interestingly, small-diameter neurons from single TRPC3 knock-out mice had 61 per cent fewer RA mechanically activated currents than WT controls (*p* < 0.01, figure 3*a*), and this loss of RA currents correlated with a doubling (97%) of the number of neurons that express IA currents (*p* < 0.01), suggesting that a population of RA current-expressing neurons had switched to an IA phenotype on deletion of TRPC3. The RA neurons had decay kinetics that were best described by a double exponential (*τ*-fast = 3.3 ms; *τ*-slow = 45 ms), whereas IA currents had decay kinetics that were best described by a mono-exponential fit. The phenotypic shift from RA to IA is therefore very clear and marked. Decay kinetics of IA neurons from WT or TRPC3 knock-out mice was not significantly different (*τ* WT = 118 ± 30 ms, *τ* TRPC3 knock-out = 107 ± 20 ms). Overall, there was no difference in the decay kinetics of RA, IA or SA currents in DRG neurons from any of the genotypes studied. When we used a defined mechanical stimulus to evoke inward currents in TRPC3 and TRPC6 knock-out mice, we found that the average peak currents were statistically indistinguishable from WT mice (figure 3*b*).

**Figure 4.**
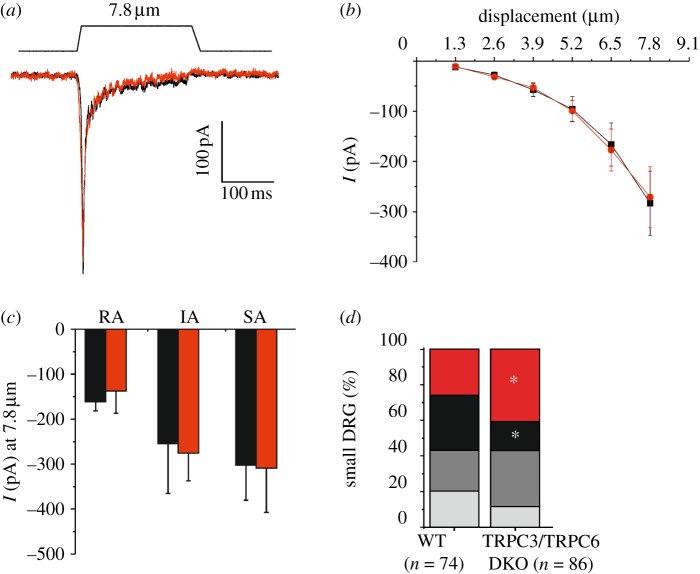
Electrophysiological characterization of sensory neurons of TRPC3/TRPC6 DKO mice. (*a*) Exemplar whole-cell voltage clamp trace from a large-diameter (narrow action potential) mouse DRG neuron in response to a 7.8 μm membrane deformation (holding potential −70 mV). All large-diameter neurons responded with a rapidly adapting current (black line, WT; red line, TRPC3/TRPC6 DKO). (*b*) The stimulus response curve of mechanically evoked peak currents in large-diameter WT (*n* = 12, black squares) and TRPC3/TRPC6 DKO (*n* = 12, red circles) mouse DRG neurons with action potential widths less than 1 ms voltage clamped at −70 mV. (*c*) The magnitude of currents evoked by a 7.8 μm stimulus in small-diameter DRG neurons with action potential width more than 1 ms (holding potential −70 mV) (black bars, WT; red bars, TRPC3/TRPC6 DKO). (*d*) Small-diameter DRG neurons had mechanically activated currents which could be classified based on their adaptation kinetics to a static mechanical stimulus: rapidly adapting (RA), intermediately adapting (IA), slowly adapting (SA). The proportion of small-diameter mouse DRG neurons expressing each current type from WT and TRPC3/TRPC6 DKO mice is shown. A significant reduction in the number of neurons displaying RA currents and an increase in number of non-responsive neurons was observed in TRPC3/TRPC6 DKO mice (*χ*^2^-test, *p* < 0.05) (red, no response; black, RA; dark grey, IA; light grey, SA).

We next examined the mechanosensitive properties of DRG neurons from TRPC3/TRPC6 DKO mice. Mechanical stimulation of large-diameter neurons (greater than 30 μm) with narrow action potentials (width of action potential is less than 1 ms) in WT and TRPC3/TRPC6 DKO mice evoked low-threshold RA mechanical currents in all cells (figure 4*a*). These inward currents in response to increasing mechanical stimuli were identical with respect to mechanical threshold of activation (WT = 3.6 ± 0.6 µm, TRPC3/TRPC6 DKO = 3.0 ± 0.4 µm, *p* > 0.05, *n* = 12), kinetics of current activation/inactivation and current magnitude in both WT and TRPC3/TRPC6 DKO mice (figure 4*a*,*b*).

When small-diameter DRG neurons with broad action potentials (width of action potential is more than 1 ms) were examined, three types of inward currents could be distinguished based on their adaptation to a 250 ms mechanical stimulus. The mean peak amplitude of the different types of currents evoked by an 8 μm distension were the same in WT and double TRPC3/TRPC6 DKO mice (figure 4*c*). However, the percentage of neurons exhibiting RA mechanosensitive currents was reduced by half (48% decrease) in neurons from DKO mice when compared with that from WT controls (*p* < 0.05), while the percentage of cells exhibiting IA and SA currents was essentially unaltered (figure 4*d*). This reduction in the number of neurons exhibiting RA currents correlated with a significant 59 per cent increase in mechanically non-responsive neurons in DKO mice (*p* < 0.05). Thus, the silencing of 50 per cent of RA mechanosensitive neurons correlated with a loss of touch sensitivity detected in behavioural assays.

### 3.4. TRPC3 and TRPC6 are present in cochlear hair cells

**Figure 5.**
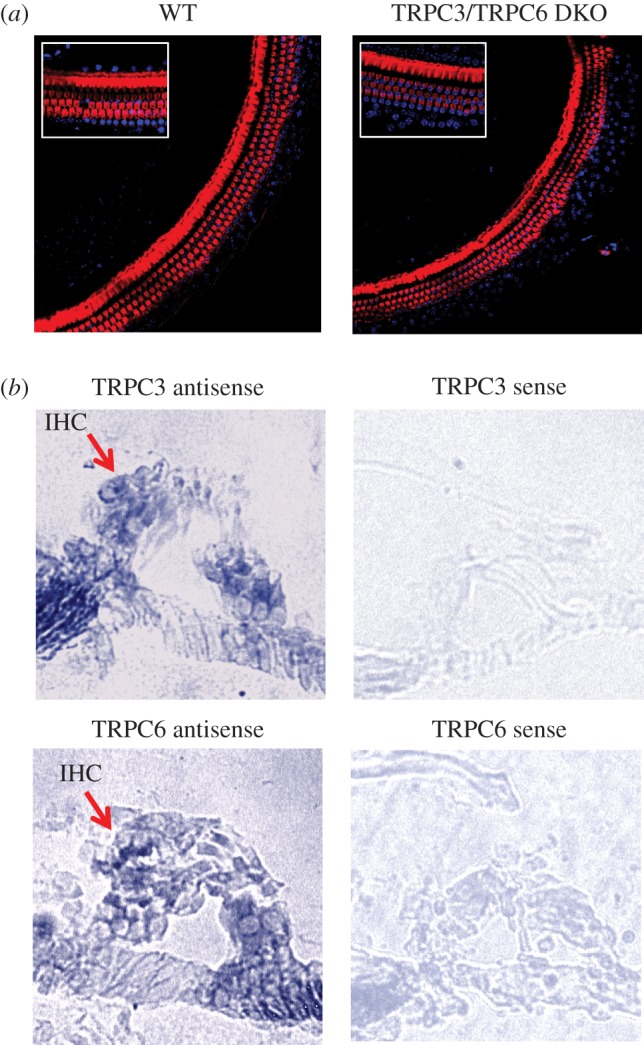
Hair cells in mouse cochlea express TRPC3 and TRPC6 and are normal in TRPC3/TRPC6 DKO mice. (*a*) Exemplar surface confocal sections of organ of Corti from WT and TRPC3/TRPC6 DKO mice (11 weeks old). Rhodamine-phalloidin (red) + nuclear DAPI (blue) staining of the cochlea showing outer hair cells and pillar cells of the basal turn (20× magnification). Inset shows 63× magnification. (*b*) *In situ* hybridization shows that TRPC3 and TRPC6 are found in the hair cells of P21 mice. Arrows indicate the location of inner hair cells (IHC). Sense probes for TRPC3 and TRPC6 produced little or no staining (see also electronic supplementary material, figure S3).

We examined the sections of cochlea from adult WT and TRPC3/TRPC6 DKO mice that were age-matched. The cochleae from both WT and TRPC3/TRPC6 DKO mice appeared grossly morphologically normal when stained with rhodamine-phalloidin to highlight stereocilia. The density of outer and inner hair cells was similar in WT mice and in TRPC3/TRPC6 DKO mice in all regions of the cochlea, including those regions associated with responses to high-frequency stimuli (figure 5*a*).

We used cochlear hair-cell cultures isolated from 2-day-old mice to examine the expression of TRPC3 and TRPC6. Cultures were grown with and without neomycin to selectively deplete hair cells. The PCR signal from TRPC3 transcripts was attenuated in hair-cell-depleted cultures showing that this channel is enriched in hair cells (see electronic supplementary material, figure S3). The PCR product from TRPC6 was unaffected by hair-cell depletion, suggesting that it may be expressed by many cell types in the cultures.

We also used *in situ* hybridization with the same probes that were validated in studies of DRG neurons from WT and knock-out mice. Figure 5*b* shows that specific cRNA probes for TRPC3 and TRPC6 hybridized to hair cells and spiral ganglion cells in cochlear sections.

### 3.5. TRPC3 and TRPC6 play a role in hearing

**Figure 6.**
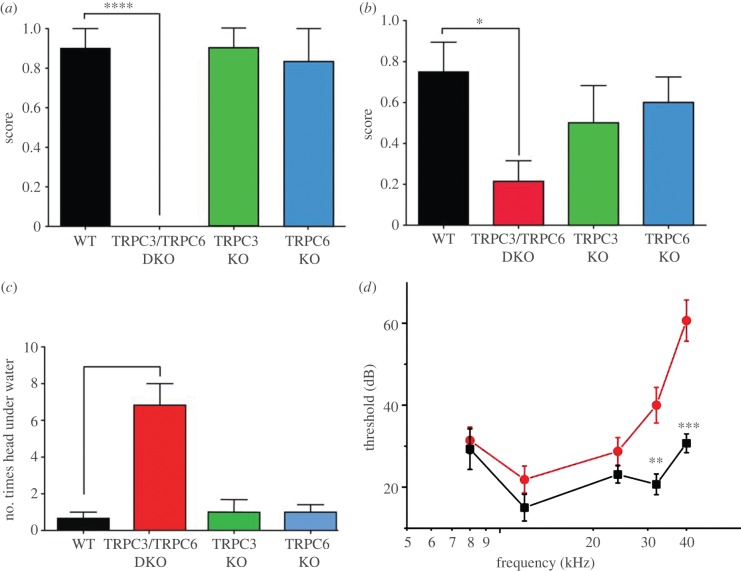
Selective hearing deficit in TRPC3/TRPC6 DKO mice. (*a*) Preyer reflex score of WT, TRPC3/TRPC6 DKO, TRPC3 KO and TRPC6 KO mice (*n* = 6 in all groups) to a click box device that emits a 90 dSB toneburst centred around 20 kHz frequency. (*b*) Reaching response, a measure of vestibular function, of WT, TRPC3/TRPC6 DKO, TRPC3 KO and TRPC6 KO mice (*n* = 6 in all groups). (*c*) Swim test ability, number of head submersions in 1 min (*n* = 6 in all groups). (*d*) Auditory brain-stem recording (ABR) response thresholds to tone bursts of WT (*n* = 8, black squares) and TRPC3/TRPC6 DKO mice (*n* = 9, red circles). Data are expressed as mean ± s.e.m. *p < 0.05; **p < 0.01; ***p < 0.001. See also electronic supplementary material, movies S1–S5 for exemplar videos of the click box and swim test.

Adult TRPC3/C6 DKO mice, but not single knock-outs appeared to have a hearing loss. We therefore examined the responses of the mouse lines to high-intensity sound. The TRPC3/TRPC6 DKO mice did not show a Preyer reflex response to loud tones centred around 20 kHz at 90 dB delivered by a custom-built click box, while single knock-out mice displayed normal responses (figure 6*a* and electronic supplementary material, movies S1–S4), indicating hearing deficits in DKOs only. Deafness syndromes are often associated with deficits in the vestibular system [5]. Trunk curl measurements and swim test behaviour showed that there was also a vestibular deficit in the DKO mice despite normal rotarod performance (see electronic supplementary material, figure S2), while there were no significant differences between WT and single knock-out animals (figure 6*b*,*c*). Analyses of other mutant mouse strains have shown that rotarod tests do not necessarily correlate with subtle vestibular deficits [28].

As TRPC3/TRPC6 DKO mice showed no behavioural responses to a high-frequency click box, we examined auditory brain-stem responses (ABRs) to pure tone stimuli in anesthetized mice [29,30]. ABR sound thresholds measured by increasing sound levels (5 dB steps) at 12, 24, 32 and 40 kHz showed that there was a significant deficit in TRPC3/TRPC6 DKO mice that was more pronounced at higher frequencies (figure 6*d*) when compared with WT mice.

### 3.6. TRPC3/TRPC6 double knock-out cochlear hair cells show deficits in mechanotransduction

**Figure 7.**
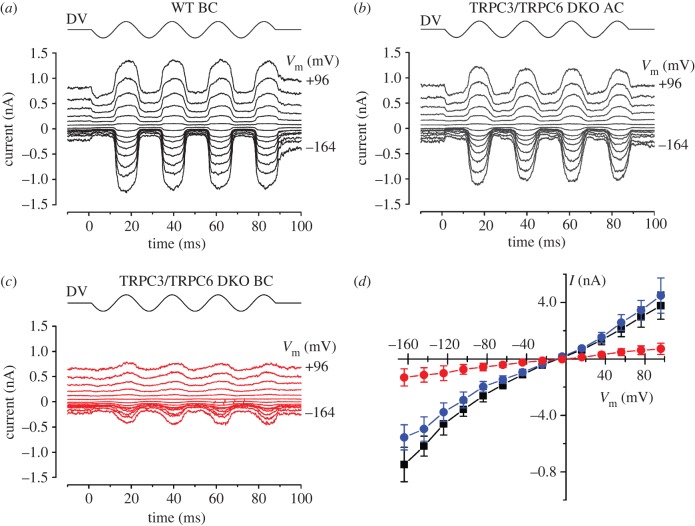
Mechano-electrical transduction in WT and TRPC3/TRPC6 DKO OHCs. (*a*–*c*) MET currents in response to 45 Hz sinusoidal force stimuli from a fluid jet in WT and TRPC3/TRPC6 OHCs. The holding potential was −84 mV and the membrane potential was stepped in 20 mV increments from −164 mV to +96 mV. Driver voltage (DV, amplitude 40 V) waveform to the fluid jet is shown above the current traces. Positive DV moves the hair bundles in the excitatory direction towards the kinocilium. Recordings in *a*–*c* are averages from two stimulus presentations each. (*a*) WT OHC, mid-basal coil P2 + 1. *C*_m_ 5.6 pF; *R*_s_ 0.80 MΩ. (*b*) TRPC3/TRPC6 DKO OHC, basal end of apical coil P2 + 2. *C*_m_ 7.4 pF; *R*_s_ 0.63 MΩ. (*c*) TRPC3/TRPC6 DKO OHC, mid-basal coil P2 + 2. *C*_m_ 6.9 pF; *R*_s_ 0.74 MΩ. (*d*) Current-voltage curves averaged from 4 WT OHCs from the basal coil (black squares), 5 apical-coil TRPC3/TRPC6 DKO OHCs (blue circles) and 8 basal-coil TRPC3/TRPC6 DKO OHCs (red circles).

Mechano-electrical transduction (MET) currents were recorded from outer hair cells (OHCs) in organotypic cochlear cultures [31] using sinusoidal force stimuli. MET currents in TRPC3/TRPC6 DKO OHCs from the basal end of the apical coil were similar in all aspects to those of WT controls from the basal coil (figure 7). In both groups, large MET currents were elicited at all potentials during the positive phase of the stimulus waveform (figure 7*a*,*b*,*d*). For example, at −104 mV, the size of the MET currents in the WT controls was −357 ± 47 pA (*n* = 4) in the OHCs and −290 ± 46 pA (*n* = 5) in the TRPC3/TRPC6 DKO OHCs situated in the apical coil (*p* > 0.05). A fraction of the MET channels was open at rest, i.e. in the absence of any stimulus, and could be seen to close in the inhibitory phase of the stimulus waveform. This fraction increased upon depolarization to positive membrane potentials, indicative of a degree of external Ca-dependent adaptation of the MET currents at hyperpolarized potentials [32]. TRPC3/TRPC6 DKO OHCs from the basal coil (figure 7*c*,*d*) had 75–80% smaller MET currents at −104 mV of −73 ± 34 pA (*n* = 8), significantly different from both other groups (*p* < 0.01). The MET currents became progressively smaller further towards the basal end of the basal coil for the TRPC3/TRPC6 DKO mutant OHCs, in contrast to the WT controls, in which this trend was not observed. Some resting MET current was nevertheless present in these OHCs (figure 7*c*). MET currents in all three groups reversed near 0 mV, as expected for non-selective cation channels.

The size of the MET currents in WT controls and TRPC3/TRPC6 DKO OHCs situated at the basal end of the apical coil was comparable to the results reported for WT OHCs studied under similar conditions [33,34]. The MET currents of the basal-coil TRPC3/TRPC6 DKO OHCs reached on average only 20–25% of the size of those of the basal-coil WT controls and the OHCs in the apical coil of the TRPC3/TRPC6 DKOs (figure 7*d*). The gradient in size of the MET currents of the TRPC3/TRPC6 DKO OHCs is thus opposite to the normal tonotypic gradient, in which currents in the basal-coil OHCs are larger than those in apical coil [35,36]. The results suggest that TRPC3 and TRPC6, either directly or indirectly, contribute to the MET currents of high-frequency, basal-coil OHCs, consistent with the high-frequency hearing loss of TRPC3/TRPC6 DKO mice. There is a tonotopic gradient in the single-channel conductance of MET currents along the cochlea, suggesting a frequency-dependent variation in subunit composition [37]. It is conceivable that either TRPC3 and/or TRPC6 are the subunits of the MET channel in basal-coil high-frequency OHCs, or that these subunits depend on TRPC3 and/or TRPC6 for their function.

### 3.7. TRPC3 and TRPC6 channels confer low-threshold mechanosensitivity on sensory neuron cell lines

Contrasting evidence exists on the mechanosensitive properties of TRPC6. It has been shown that TRPC6 can produce inward currents evoked by mechanical or osmotic stimuli in TRPC6-expressing cells [38]. However, there are other reports that show that overexpression of TRPC6 does not confer mechanosensitivity on cells [39]. These differences may be reconciled in part by the demonstration that indirect activation of TRPC6 can occur through mechanosensitive G protein-coupled receptors (GPCRs) such as the angiotensin AT1 receptor that are expressed only in certain cell types [4]. Currently there is no evidence that TRPC3 confers mechanosensitivity on any cell types.

**Figure 8.**
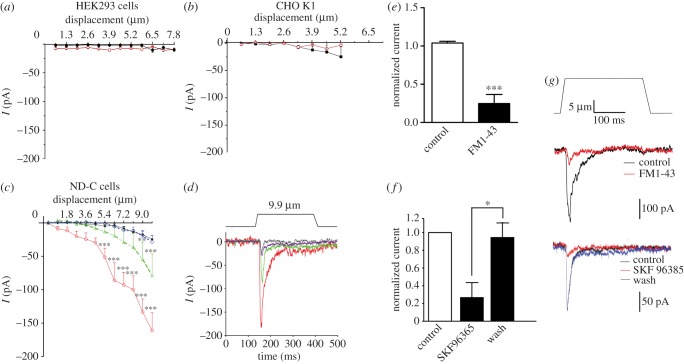
Heterologous expression of TRPC3 with or without TRPC6 confers mechanosensitivity on some cell lines. Cells were transfected with constructs encoding TRPC3-IRES-GFP + TRPC6-IRES-DsRed2, TRPC3-IRES-GFP alone, TRPC6-IRES-DsRed2 alone or IRES-GFP and/or IRES-DsRed2. Cells were selected based on their fluorescence and voltage-clamped at −70 mV in whole-cell configuration. The magnitude of mechanically evoked currents in (*a*) HEK293a cells, (*b*) CHO K1 ((*a*,*b*): black dots, empty vector (*n* = 9); red circles, TRPC3/TRPC6 (*n* = 9)) or (*c*) ND-C cells, a sensory neuron cell line is presented (black dots, empty vector (*n* = 33); red circles, TRPC3/TRPC6 (*n* = 11); green triangles, TRPC3 (*n* = 12); blue diamonds, TRPC6 (*n* = 13)). (*d*) Exemplar traces from (*c*) induced by a 9.9 μm membrane displacement (top plot: movement of the probe). ND-C cell were transfected with TRPC3 and TRPC6 and voltage-clamped at −70 mV in whole-cell configuration. Mechanically activated currents were elicited by an approximately 14 µm displacement of a glass probe at 10 μm distance from the cell (black lines, empty vector; red lines, TRPC3/TRPC6; green lines, TRPC3; blue lines, TRPC6). (*e*) Normalized current amplitudes from three cells before, during and 30 s after application of SKF96365. (*f*) Normalized current amplitudes (*n* = 7) before and after application of FM1-43. (*g*) Representative current traces before (black), during (red) and 30 s after (blue) application of 100 μM SKF96365 or 15 μM FM1-43. Top plot shows movement of displacement of glass probe. Data represent mean ± s.e.m. **p < 0.01; ***p < 0.001 (see also electronic supplementary material, figure S4).

We examined the mechanosensitivity of cell lines over-expressing TRPC3, TRPC6 or both channels using the same methods that demonstrated a deficit in TRPC3/TRPC6 DKO sensory neurons. When the channels were over-expressed either singly or in combination in HEK293a cells or chinese hamster ovary (CHO) cells, no mechanically evoked inward currents could be detected (figure 8*a*,*b*). However, when the two channels were expressed in ND-C cells [40], a sensory neuron-derived cell line, RA currents could be detected even after small mechanical displacements (approx. 1 µm), while no currents were evoked in control transfected ND-C cells at membrane displacements less than 8 µm (figure 8*c*,*d*). Mechanical stimulation of TRPC3-expressing cells evoked an inward current that developed within milliseconds and increased in size with the strength of the mechanical distension (figure 8*c*,*d*). The decay kinetics of the mechanically activated currents in TRPC3-expressing cells was best described by a mono-exponential fit (*τ* = 37.2 ± 6.7 ms, *n* = 12). Co-expression of TRPC6 with TRPC3 shifted the stimulus response curve to the left, resulting in a lower threshold of activation and increased currents in response to mechanical stimuli. Additionally, the decay kinetics of mechanically activated currents in TRPC3 and TRPC6-expressing cells were significantly faster compared with cells expressing TRPC3 alone (*τ* = 20.1 ± 1.2 ms, *n* = 10, *p* < 0.05). TRPC6 expression alone did not confer increased mechanosensitivity on ND-C cells (figure 8*c*,*d*). We confirmed that the expressed currents were potentially due to TRPC3 and TRPC6 expression, using SKF96365, a blocker with some selectivity for TRPC channels, that reversibly blocked mechanically gated currents in ND-C cells transfected with TRPC3 and TRPC6 (figure 8*f*,*g*). The fluorescent styryl dye FM1-43 is a permeant blocker of mechanosensitive channels in both the cochlea and sensory neurons. We found that TRPC3/TRPC6-dependent mechanosensitive inward currents were also blocked by micromolar FM1-43 concentrations (figure 8*e*,*g*).

Overall, these data show that the expression of the combination of TRPC3 and TRPC6 results in the formation of mechanosensitive channel activity that was different in kinetics from TRPC3 alone, consistent with some form of interaction that is dependent on the correct cellular context. It is noteworthy that there are many examples of transcripts that are only functionally expressed in a particular cell type, an extreme example being Na_v_1.9, a sodium channel that can be expressed only in sensory neurons [41]. The expression of TRPC3 in ND-C cells (but not in HEK293a cells or CHO cells) resulted in mechanically gated inward currents. The expression of TRPC6 in the cell lines that we tested did not result in mechanically gated inward currents, although other groups have described mechanosensitive activity conferred by TRPC6 expression in HEK293 cells. Spassova *et al*. [38] showed that mechanical or osmotic stimuli could produce inward currents in cells expressing TRPC6 that were blocked by GsMTx-4. This toxin is also able to block the activity of Piezo channels [42]. It has been proposed that TRPC6 is not intrinsically mechanosensitive [39], but requires co-expression with a Gq-coupled receptor [43]. However, we found that co-expression of TRPC6 with the angiotensin receptor AT1 did not confer mechanosensitivity on CHO cells (see electronic supplementary material, figure S4*b*).

We have shown a potentiation of TRPC3-mediated mechanically evoked currents by TRPC6, and it is possible that TRPC6 performs a similar function for other TRP channels, or is itself a significant mechanotransducer in the correct cellular context. The FAM38a and b proteins (renamed Piezo1 and 2) confer mechanosensitivity on cell lines, producing rapidly inactivating cation-selective channel activity. Piezo2 is expressed in a subset of DRG sensory neurons, and siRNA-mediated gene knockdown causes a silencing of about 75 per cent of the sensory neurons that express RA mechanosensitive channels [2]. Whether this population of silenced neurons overlap with, or are distinct from the neurons that are silenced in the TRPC3/TRPC6 DKO is unknown. The time constant for inactivation of mechanically activated currents in ND-C cells expressing TRPC3/6 is different from Piezo1-mediated mechanically activated currents in N2A cells [2] (τ = 20.1 ± 1.2 versus 15.3 ± 1.5 *p* < 0.01) and is also different from the kinetics of endogenous mechanosensitive ion channels in DRG. However, the pharmacology of channel block by SKF96365 is consistent with some role for TRPC3 and TRPC6 in mechanosensory channel activity.

## 4. Discussion

Touch and hearing involve the activation of mechanically sensitive cation-selective ion channels. Somatosensory neurons with narrow action potentials are intrinsically mechanosensitive [26]. A range of end organs (e.g. Merkel cells) are linked to the detection of mechanical stimuli and are intimately associated with such A-fibre sensory neurons [44]. Those neurons that mainly respond to tissue damage express three classes of mechanically gated ion channel that can be distinguished pharmacologically [45]. The SA currents are associated with noxious mechanosensation [45]. In the present study, we have found that deleting both TRPC3 and TRPC6 has no effect on mechanically gated currents in large-diameter sensory neurons with narrow action potentials. However, a set of small-diameter sensory neurons, that express RA mechanosensitive channels are silenced by removal of these channels. A partial loss of mechanosensitivity is also a consequence of channel deletion. Recently, unmyelinated small-diameter neurons that are responsive to von Frey hairs have been linked to mechanical hyperalgesia [46].

Hearing and balance are dependent on hair cells within the inner ear. The mechanically sensitive part of the hair cell, the stereociliary bundle, comprises 50–100 individual stereocilia. Sound causes a small displacement of the bundle, leading to the opening of calcium-permeant ion channels. Hair cells are arranged along the length of the cochlea and vary in their frequency sensitivity, with cells responding to the highest frequencies found at the base of the cochlea. The kinetics of the mechanically activated channels of cochlear OHCs also vary with an increase in single channel conductance and channel number from low to high frequencies [37]. The mechanically activated channels are calcium-permeant, linked to their Ca^2+^-dependent adaptation and can be blocked by Gd^3+^, ruthenium red, amiloride and aminoglycoside antibiotics [47–50]. FM1-43 (a fluorescent styryl dye) is a permeant blocker of channels in both cochlear hair cells and sensory neurons [51,52], and blocked TRPC3/C6 mechanosensitive channel activity in ND-C cells.

TRP channels have been implicated in mechanosensation in the cochlea. TRPV4, which can associate with TRPC channels, is involved in mechanical hyperalgesia and is found in the ear, although mainly in the stria vascularis [53], and TRPV4 knock-out mice have a late-onset hearing deficit [12]. However, an osmoregulatory role is likely because of the expression in the stria rather than hair cells, and the slow activation that is inconsistent with mechanical transduction [12]. Varitint-waddler mice exhibit deafness and circling behaviour [54]. The mutant gene was mapped to TRPML3 [55], and a gain-of-function mutation causes TRPML3 to act as an inwardly rectifying cation channel in OHCs of homozygous Varitint-waddler mutant mice [56,57]. The resulting leak conductance through the TRPML3 channels, whose properties are clearly distinct from the hair cell transducer channel [57], leads over time to hair cell degeneration and deafness [54]. TRPA1 is expressed in the sensory epithelium of the mouse utricle and has large numbers of ankyrin repeats that have been suggested to be a possible gating spring, but activators of TRPA1 have no affect on hair-cell transduction and TRPA1 knock-out mice have no obvious hearing deficits, although ABR to low-frequency sound are potentiated in the absence of TRPA1 [13,58].

Canonical TRP channels were the first mammalian homologues of the *Drosophila* TRP channel to be identified and are all present in the cochlea [59]. Interestingly, both TRPC3 and TRPC6 are known to interact not only with each other, but also with BK channels that regulate K^+^ fluxes indirectly through altered intracellular levels of calcium [60]. BK channels are present in inner hair cells and encode a fast-activating outward current [61,62]. BK-channel mouse knock-outs show increased resistance to noise-induced hearing loss, but surprisingly few other deficits suggest that these channels are less significant for hearing in mammals than in birds and reptiles, in which they play a role in frequency tuning [63].

The expression of TRPC3 in ND-C cells (but not in HEK293a cells) or CHO cells resulted in mechanically gated inward currents. The expression of TRPC6 in the cell lines that we tested did not result in mechanically gated inward currents, although other groups have described a very slowly developing (seconds or minutes) mechanosensitive activity conferred by TRPC6 expression in HEK293 cells. We have shown a potentiation of TRPC3-mediated mechanically evoked currents by TRPC6, and it is possible that TRPC6 performs a similar function for other TRP channels, or is itself a significant mechanotransducer in the correct cellular context. It is noteworthy that deletion of TRPC6 leads to an upregulation of TRPC3 expression in smooth muscle cells [64].

If TRPC3 is an intrinsically mechanosensitive channel as suggested by the heterologous expression studies, then why do TRPC3 knock-out mice appear behaviourally normal in terms of responses to light touch and click box tone bursts? In fact, there is a clear deficit in terms of DRG neurons expressing RA currents in TRPC3 knock-out mice. The cells that lose RA currents are replaced by cells that express IA currents. These observations can be most simply explained if there is a third component of mechanosensory complexes, that interacts with TRPC6 to produce IA currents in DRG sensory neurons. In this scheme, TRPC3-expressing cells convert the intermediate adapting channel properties of the unknown component to RA kinetics. A large network of TRPC-interacting proteins has been annotated, providing candidates for such a component [25]. As this component produces IA currents in this speculative scheme, it is unlikely to be Piezo2. There are many other possible explanations, including TRP-dependent regulation of Piezo expression. However, as the number of DRG neurons that are mechanosensitive is larger than the number that expresses Piezo2 [2], it is likely that there is more than one mechanotransducing mechanism in play.

Our results provide evidence that while TRPC3 and TRPC6 play a role in mechanotransduction, they are not sufficient to make a mechanotransduction complex alone, because overexpression of TRPC3 and TRPC6 does not result in mechanically activated currents in some cell lines. Indirect activation by lipid mediators released by mechanosensitive GPCRs or trafficking into the membrane could contribute to mechanically gated currents. In sensory neurons, the voltage-gated sodium channel Na_v_1.8 is only expressed functionally on cell membranes in the presence of P11, an Annexin molecule, and may be overexpressed in other cell lines where it fails to produce sodium channel activity at the membrane [65]. TMC1 confers mechanosensitivity only on cochlear hair cells and is another potential mechanotransducing channel that is inactive when expressed in a variety of cell lines [6].

It is tempting to speculate that different repertoires of TRP channels contribute to mechanosensation in an analogous way to TRPV1, TRPM8 and TRPA1 that contribute to temperature-sensing [44]. In a subset of sensory neurons, RA mechanically gated currents clearly require TRPC3 and TRPC6. TRPA1 channels are required for SA mechanosensory channels that are linked to noxious mechanosensation in a different set of sensory neurons [14]. The published data on TRP channels and mechanosensation that are consistent with the idea of a combinatorial TRP code for mechanosensation are collated in a table in supplementary material (see electronic supplementary material, table S1). These data could help explain the failure of monogenic screens to identify candidate mechanotransducing channels for hearing or touch, as multiple related genes would need to be ablated to see a substantial phenotype. It is striking that only a subset of sensory neurons and cochlear hair cells lose mechanosensitivity in TRPC3/TRPC6 DKO mice. However, it could also be argued that mechanotransducing channels may be so important in various systems that embryonic lethality results from their ablation.

In the cochlea, calcium homeostasis is a key element in the functioning of the calcium permeant mechanosensory channels that underlie hearing [5,66]. It is possible that TRPC3 and TRPC6 are necessary for regulating intracellular calcium levels to enable hair-cell function, and that other channels—for example TMC, Piezo or other proteins—are the mechanotransducing channels [2,6]. Nonetheless, mechanotransduction in RA hair cells and high-frequency hair cells both require the expression of TRPC3 and TRPC6, and touch and hearing are compromised in their absence. The ability to express RA mechanotransducing channels within cell lines should now enable us to identify other components that are responsible for TRPC3/TRPC6-dependent mechanotransduction in NDC cells. The present findings thus increase the known functional repertoire of TRPC channels and provide an interesting mechanistic link between two related sensory modalities, hearing and touch.

## 5. Methods

### 5.1. Generation of TRPC3/TRPC6 double knock-out mice

TRPC3/TRPC6 DKO mice were generated by Birnbaumer *et al*. (also see electronic supplementary material, figure S3). TRPC3/TRPC6 DKO mice were crossed with C57BL/6 mice to generate heterozygous TRPC3^+/−^ TRPC6^+/−^ mice. Heterozygous mice were then crossed together to create a number of genotypes, including homozygous TRPC3 knock-out mice, homozygous TRPC6 knock-out mice, homozygous TRPC3/TRPC6 DKO mice and WT controls.

### 5.2. Behavioural studies

Mechanical sensitivity was assessed using von Frey hairs using the up-down method described by Chaplan *et al*. [67] to calculate the 50 per cent withdrawal threshold. Mechanical sensitivity was also assessed using the cotton swab test as described [20], briefly the cotton swab was ‘puffed out’ so that the cotton head was more than three times the normal size and a less than 1-s stroke was applied along the plantar paw surface five times, alternating between paws with a 10-s interval between applications. The number of paw withdrawals was recorded and averaged over two trials. Noxious mechanical sensitivity was assessed using Randall–Selitto apparatus to apply increasing pressure to the tail. Thermal sensitivity was measured by calculating mean paw withdrawal latency from a radiant heat source over an average of at least three trials.

Hearing function was assessed using a custom-built click box, (MRC Institute of Hearing Research) held 30 cm above the mouse which delivered a calibrated 20 kHz toneburst at an intensity of 90 dB SPL. The presence or absence of an ear flick response (Preyer reflex) was recorded. If the animal responded well with a full response, a score of 1 was given, partial response was scored with 0.5 and if the animal did not respond at all, a score of 0 was given [68]. Vestibular function was assessed with the trunk curl test. The mouse was held in the air by the tail 5 cm away from a horizontal surface for 5 s. If the mouse reached out with its forelimbs towards the platform, it received a score of 1. If the mouse curled towards its belly, it received a score of 0. If the mouse curled only slightly it received a score of 0.5 [69]. The swim test was performed in at least 15 cm of 28°C water. Mice were placed into the water and observed for 1 min before being retrieved and dried under a heat lamp. The swim test was videotaped and manually scored for abnormal swimming behaviours such as head submersions. The experimenter was blind to the genotypes of the mice during the experiment and scoring.

### 5.3. Auditory brainstem response

Mice between four and 12 weeks were anesthetized with ketamine-medetomidine cocktail (50 mg ketamine, 0.415 mg medetomidine in 4.1 ml saline), 0.01 ml g^–1^ body weight. Electrodes were placed at the vertex and the mastoid, with a ground near the tail. TDT System 3 equipment was used to generate the stimuli and acquire the evoked response. Tone pips were presented through a closed-field speaker at 25 s^–1^ at each frequency. Pure tone stimuli were generated at 8, 12, 24, 32 and 40 kHz. The sound pressure level was varied in 5 dB steps from 10 to 90 relative dB. The data was converted using Matlab, and evoked potential wave forms were visually inspected to obtain a threshold value.

### 5.4. Fluorescence microscopy

Mice were killed directly after functional testing and cochleae excised from bullae. Fixative was perfused directly into the cochlea by creating a small opening at the apex of the cochlea and rupturing the bone between the round and oval window. Cochleae were fixed in 4 per cent paraformaldehyde for 90 min and then decalcified in 4.13 per cent EDTA (pH 7.3). Organ of Corti strips (1/2 turns) were carefully dissected, categorized into apical or basal, and incubated in 0.5 per cent Triton X-100 for 20 min to permeabilize plasma membranes. Tissue was then incubated in phalloidin conjugated to rhodamine fluorophore 1 μg ml^–1^ in phosphate buffered saline (PBS) with 0.15 per cent Triton X-100 for 80 min at room temperature. Wholemounts were washed in PBS for four times and mounted on multispot slides with Vectashield containing DAPI (4′,6-diamidino-2-phenylindole) to counterstain nuclei. Preparations were examined and imaged using a Zeiss Meta confocal laser scanning microscope. Digital images were exported as TIFF or JPEG files.

### 5.5. *In situ* hybridization

TRPC3 or TRPC6 knock-out or WT adult mice were perfused and the DRGs removed and fixed in 4 per cent PFA in PBS followed by post-fixation in 30 per cent sucrose. Cochlea were removed from P21 mice after perfusion and fixed in 4 per cent paraformaldehyde (PFA) in PBS followed by post-fixation in 30 per cent sucrose. After post-fixation, cochlea were decalcified in 120 mM EDTA for 24–48 h or until the bone was soft. 12 µm frozen sections of DRG or cochlea were hybridized with digoxigenin-labelled cRNA probes targeted to either TRPC3 or TRPC6. The cRNA probes were generated from cDNA fragments of around 200–300 bp length (TRPC3 position nt +2985 to +3185; TRPC6 position nt +2384 to +2618) or 900–1000 bp length (TRPC3 position nt +1863 to +2871; TRPC6 position nt +1570 to +2567). The cDNA fragments were cloned into the pGEM-T-Easy cloning vector (Promega) and labelled with digoxigenin (DIG) by *in vitro* transcription using the DIG RNA labelling Kit (SP6/T7, Roche). The antisense cRNA was used for the detection of mRNAs.

### 5.6. Culture of dorsal root ganglion neurons

Adult mice DRG neurons were dissected out and subsequently digested in an enzyme mixture containing Ca^2+^- and Mg^2+^-free Hanks' balanced salt solution (HBSS), 5 mM 4-(2-hydorxyethyl)piperazine-l-ethanesulphonic acid (HEPES), 10 mM glucose, collagenase type XI (5 mg ml^−1^), dispase (10 mg ml^−1^) for 1 h prior to mechanical trituration in Dulbecco's modified Eagle's medium (DMEM) + 10 per cent heat-inactivated foetal bovine serum (FBS). Cells were then centrifuged for 5 min at 800 r.p.m. and resuspended in DMEM containing 4.5 g l^−1^ glucose, 4 mM l-glutamine, 110 mg l^−1^ sodium pyruvate, 10 per cent FBS, 1 per cent penicillin–streptomycin (10 000 i.u. ml^−1^), 1 per cent Glutamax, 125 ng ml^−1^ nerve growth factor (NGF) and 50 ng ml^−1^ Neurotrophin-4 (NT-4) and plated on 35 mm dishes coated with poly-l-lysine (0.01 mg ml^−1^) and laminin (0.02 mg ml^−1^). Cultures were kept at 37°C in 5 per cent CO_2_. Neurons were used up to 2 days after plating.

### 5.7. Cell culture

ND-C and HEK293a cells were cultured in DMEM supplemented with 10 per cent foetal calf serum (FCS); CHO K1 cells were cultured in DMEM/F-12 (1 : 1) supplemented with 10 per cent FCS. One day before transfection, cells were seeded to give 40–60% confluency on the day of transfection. Plasmid DNA was transiently transfected into the cells using Lipofectamine 2000 (Invitrogen, UK) in a ratio of 1 μg DNA: 2.5 μl Lipofectamine 2000, according to the manufacturer's instructions. Electrophysiology recordings were made 24–48 h post-transfection.

### 5.8. Electrophysiology

Neurons whose cell bodies were not in contact with those of other neurons and ND-C cells tagged with fluorescent proteins were selected for recording. Currents were recorded using Axopatch 200B and Multiclamp 700 amplifiers (Axon Instruments, Molecular Devices Inc.). Pipettes were pulled from borosilicate glass capillaries with a P-97 puller (Sutter Instrument Co.) and had resistances of 2–4 MΩ when filled with the pipette solution. Currents were digitized with the Digidata 1322A and 1440A data acquisition systems (Axon Instruments, Molecular Devices Inc.). Data were recorded and stored using Clampex 8.1, 9 and 10 (Axon Instruments, Molecular Devices Inc.). Currents were low-pass filtered at 2–5 kHz and sampled at 10–12.5 kHz. Capacity transients were cancelled; however, series resistances were not compensated. Voltages were not corrected for liquid junction potentials. Recordings were performed at room temperature. Offline analysis, fits and statistics were performed using Clampfit 9.0 and 10 (Axon Instruments, Molecular Devices Inc.), Office Excel (Microsoft Corp.), SigmaPlot 8 (Systat Software Inc.) and Prism (GraphPad software). Recordings were carried out in the perforated patch configuration. The pipette solution contained (in mM) 110 CH_3_COOK, 30 KCl, 5 NaCl, 1 MgCl_2_ and 10 HEPES (pH corrected to 7.35 using KOH, osmolarity set to 310–320 mOsm with sucrose). A total of 250 μg ml^−1^ of fresh amphotericin B was added to this solution prior to recording. The bath solution contained (in mM): 140 NaCl, 4 KCl, 2 CaCl_2_, 1 MgCl_2_, 5 glucose and 10 HEPES (pH 7.4 adjusted using NaOH; osmolarity set to 310–320 mOsm with sucrose).

### 5.9. Mechanical stimulation

Mechanical stimulation of cell bodies was achieved using a heat-polished glass pipette (tip diameter approximately 2 μm), controlled by a piezo-electric crystal drive (Burleigh LSS-3000 series and Siskiyou MXPZT-300 series), positioned at an angle of about 70° to the surface of the dish. The probe was positioned so that a 10 μm movement did not visibly contact the cell but that an 11 μm stimulus produced an observable membrane deflection. The probe was moved at a speed of approximately 1 μm ms^–1^, and the stimulus was applied for 250 ms. A series of mechanical steps in 1 (ND-C cells, HEK cells or CHO cells) or 2 μm (DRG neurons) increments were applied. DRG neurons that did not respond to mechanical stimulations of up to 12 μm were considered mechanically insensitive. Criteria for classifying adaptation kinetics of mechanosensitive currents were as follows. RA currents had a decay kinetic that was best described by a bi-exponential fit. IA currents had a decay kinetic that was best described by a mono-exponential fit. Activation of SA currents was slow in comparison to RA and IA and could be described by a mono-exponential fit. Minimal decay of the current was observed during the 250 ms mechanical stimulus (less than 20%). Taus were obtained by fitting of mean data (*n* = 10). Thresholds of activation for mechanically activated currents were determined as an inward current larger than 10 pA.

### 5.10. TRPC3-IRES-AcGFP1 and TRPC6-IRES-DsRed2 expression plasmids

The expression constructs were designed to include internal ribosome entry sites (IRES) so that the green fluorescent protein, AcGFP1, was translated in cells expressing TRPC3 and the red fluorescent protein, DsRed2, was translated in cells expressing TRPC6. TRPC3 (NM_003305) was polymerase chain reaction (PCR)-amplified from human cDNA clone SC310083 (OriGene) using the forward primer (5′ CGG AAT TCC TAC TGA TTA GGT CCA TGG AG) and the reverse primer (5′ CGG GAT CCA GAG CTA ACT TTT AAA GGT TC) and Phusion High-Fidelity DNA Polymerase (New England Biolabs). This 2716 bp fragment was digested with *Eco*RI and *Bam*HI and ligated into pIRES2-AcGFP1 (Clontech).

The TRPC6-IRES-DsRed2 construct was prepared in two stages. Firstly, the polio IRES-DsRed2 fragment (1487 bp) was PCR-amplified from vector POLRED1 [70] using the forward primer (5′ CCG CTC GAG CGG CCG CTA GCG CTA CCG GAC TC) and the reverse primer (5′ CTA GAG GGC CCC TAC AGG AAC AGG TGG TGG C) and Phusion High-Fidelity DNA Polymerase (New England Biolabs). This fragment was digested with *Xho*I and *Apa*I and ligated into pcDNA3 (Invitrogen) to give pcDNA3-POLRED. Next, TRPC6 (NM_004621) was PCR-amplified from human cDNA clone SC310040 (OriGene) using the forward primer (5′ CGC GGA TCC TCG GGC GTT CCC GCC ATG AGC CAG) and the reverse primer (CCG GAA TTC ACG TTT TCT TGT TTA AAA GGT GG). This 2938 bp fragment was digested with *Bam*HI and *Eco*RI and ligated into pcDNA3-POLRED. The coding sequence of all constructs was sequenced entirely.

### 5.11. Heterologous expression of TRPC3 and TRPC6 in ND-C and CHO K1 cell lines

ND-C cells [40] were cultured in DMEM supplemented with 10 per cent FCS; CHO K1 cells were cultured in DMEM/F-12 (1 : 1) supplemented with 10 per cent FCS. One day before transfection, cells were seeded to give 40–60% confluency on the day of transfection. Plasmid DNA was transiently transfected into the cells using Lipofectamine 2000 (Invitrogen) in a ratio of 1 µg DNA: 2.5 µl Lipofectamine 2000 according to the manufacturer's instructions. Electrophysiological recordings were made 24–48 h post-transfection.

### 5.12. Mechano-electrical transduction current recording

MET currents were recorded from OHCs in organotypic cochlear cultures [31] made at postnatal day 2 (P2) and maintained *in vitro* for one or two days. Extracellular solution was continuously perfused and contained (in mM): 135 NaCl, 5.8 KCl, 1.3 CaCl_2_, 0.9 MgCl_2_, 0.7 NaH_2_PO_2_, 2 Na-pyruvate, 5.6 d-glucose, 10 HEPES, with amino acids and vitamins for Eagle's minimum essential medium (MEM) added from concentrates (Invitrogen). The pH was adjusted to 7.5 with 1 M NaOH and the osmolality was about 306 mmol kg^−1^. The organs of Corti were observed with an upright microscope (Olympus BX50WI, Japan) with Nomarski optics.

Mechano-electrical transducer currents were elicited using fluid-jet stimulation (45 Hz sinewaves filtered at 1.0 KHz, 8-pole Bessel) and recorded at room temperature (20–22°C) under whole-cell voltage clamp (Cairn Optopatch, UK) as previously described [33]. The holding potential was −84 mV and amplitude of the driver voltage to the fluid jet was 40 V to generate forces sufficiently large to elicit near-saturating transducer currents in WT control cells. Patch pipettes (resistance in the bath 1.9–2.5 MΩ) were pulled from soda glass capillaries and coated with wax. Intracellular solutions contained (in mM): 137 CsCl, 2.5 MgCl_2_, 1 EGTA-CsOH, 2.5 Na_2_ATP, 10 Na_2_phosphocreatine, 5 HEPES; pH adjusted to 7.3 with 1 M CsOH; osmolality 293 mmol kg^−1^. Data were filtered at 2.5 kHz, sampled at 25 kHz and stored on computer for offline analysis using Origin software. No correction was applied for the voltage drop across the series resistance after compensation of 60–80%, which was at most 5 mV at extreme potentials. Membrane potentials were corrected for a –4 mV liquid junction potential between pipette and bath solutions. Averaged MET currents of OHCs are reported as mean ± s.e.m. Statistical comparisons were made using one-way ANOVA followed by the Tukey–Kramer multiple comparisons test. *p* < 0.05 was used as the criterion for statistical significance.

### 5.13. Statistical analysis

Data are expressed as mean ± s.e.m. Measurements were compared using Student's *t*-test, one-way ANOVA or two-way ANOVA followed by Bonferroni's analysis. A *p*-value < 0.05 was considered to be statistically significant. For comparison of the proportion of neurons displaying different kinetics of mechanically activated currents, a two-tailed χ^2^-test was used.

## Supplementary Material

Electronic supplementary material

## References

[1] Arnadottir J Chalfie M 2010 Eukaryotic mechanosensitive channels. Annu. Rev. Biophys. 39, 111–137 10.1146/annurev.biophys.37.032807.125836 (doi:10.1146/annurev.biophys.37.032807.125836) 20192782

[2] Coste B Mathur J Schmidt M Earley TJ Ranade S Petrus MJ Dubin AE Patapoutian A 2010 Piezo1 and Piezo2 are essential components of distinct mechanically activated cation channels. Science 330, 55–60 10.1126/science.1193270 (doi:10.1126/science.1193270) 20813920 PMC3062430

[3] Noel J 2009 The mechano-activated K^+^ channels TRAAK and TREK-1 control both warm and cold perception. EMBO J. 28, 1308–1318 10.1038/emboj.2009.57 (doi:10.1038/emboj.2009.57) 19279663 PMC2683043

[4] Patel A Sharif-Naeini R Folgering JRH Bichet D Duprat F Honoré E. 2010 Canonical TRP channels and mechanotransduction: from physiology to disease states. Pflugers Arch. 460, 571–581 10.1007/s00424-010-0847-8 (doi:10.1007/s00424-010-0847-8) 20490539

[5] Dror AA Avraham KB 2010 Hearing impairment: a panoply of genes and functions. Neuron 68, 293–308 10.1016/j.neuron.2010.10.011 (doi:10.1016/j.neuron.2010.10.011) 20955936

[6] Kawashima Y 2011 Mechanotransduction in mouse inner ear hair cells requires transmembrane channel-like genes. J. Clin. Invest. 121, 4796–4809 10.1172/JCI60405 (doi:10.1172/JCI60405) 22105175 PMC3223072

[7] Gong Z 2004 Two interdependent TRPV channel subunits, inactive and Nanchung, mediate hearing in *Drosophila*. J. Neurosci. 24, 9059–9066 10.1523/JNEUROSCI.1645-04.2004 (doi:10.1523/JNEUROSCI.1645-04.2004) 15483124 PMC6730075

[8] Tracey WD Jr Wilson RI Laurent G Benzer S 2003 *painless*, a *Drosophila* gene essential for nociception. Cell 113, 261–273 10.1016/S0092-8674(03)00272-1 (doi:10.1016/S0092-8674(03)00272-1) 12705873

[9] Walker RG Willingham AT Zuker CS 2000 A *Drosophila* mechanosensory transduction channel. Science 287, 2229–2234 10.1126/science.287.5461.2229 (doi:10.1126/science.287.5461.2229) 10744543

[10] Chatzigeorgiou M 2010 Specific roles for DEG/ENaC and TRP channels in touch and thermosensation in *C. elegans* nociceptors. Nat. Neurosci. 13, 861–868 10.1038/nn.2581 (doi:10.1038/nn.2581) 20512132 PMC2975101

[11] Kang L Gao J Schafer WR Xie Z Xu XZ 2010 *C. elegans* TRP family protein TRP-4 is a pore-forming subunit of a native mechanotransduction channel. Neuron 67, 381–391 10.1016/j.neuron.2010.06.032 (doi:10.1016/j.neuron.2010.06.032) 20696377 PMC2928144

[12] Tabuchi K Suzuki M Mizuno A Hara A 2005 Hearing impairment in TRPV4 knockout mice. Neurosci. Lett. 382, 304–308 10.1016/j.neulet.2005.03.035 (doi:10.1016/j.neulet.2005.03.035) 15925108

[13] Kwan KY Allchorne AJ Vollrath MA Christensen AP Zhang D-S Woolf CJ Corey DP 2006 TRPA1 contributes to cold, mechanical, and chemical nociception but is not essential for hair-cell transduction. Neuron 50, 277–289 10.1016/j.neuron.2006.03.042 (doi:10.1016/j.neuron.2006.03.042) 16630838

[14] Vilceanu D Stucky CL 2010 TRPA1 mediates mechanical currents in the plasma membrane of mouse sensory neurons. PLoS ONE 5, e12177 10.1371/journal.pone.0012177 (doi:10.1371/journal.pone.0012177) 20808441 PMC2922334

[15] Kerstein PC del Camino D Moran MM Stucky CL 2009 Pharmacological blockade of TRPA1 inhibits mechanical firing in nociceptors. Mol. Pain 5, 19 10.1186/1744-8069-5-19 (doi:10.1186/1744-8069-5-19) 19383149 PMC2681449

[16] Abramowitz J Birnbaumer L 2009 Physiology and pathophysiology of canonical transient receptor potential channels. FASEB J. 23, 297–328 10.1096/fj.08-119495 (doi:10.1096/fj.08-119495) 18940894 PMC2630793

[17] Folgering JH Sharif-Naeini R Dedman A Patel A Delmas P Honoré E. 2008 Molecular basis of the mammalian pressure-sensitive ion channels: focus on vascular mechanotransduction. Prog. Biophys. Mol. Biol. 97, 180–195 10.1016/j.pbiomolbio.2008.02.006 (doi:10.1016/j.pbiomolbio.2008.02.006) 18343483

[18] Sharif-Naeini R Folgering JHA Bichet D Duprat F Delmas P Patel A Honoré E 2010 Sensing pressure in the cardiovascular system: Gq-coupled mechanoreceptors and TRP channels. J. Mol. Cell. Cardiol. 48, 83–89 10.1016/j.yjmcc.2009.03.020 (doi:10.1016/j.yjmcc.2009.03.020) 19345226

[19] Yasuda N 2008 Conformational switch of angiotensin II type 1 receptor underlying mechanical stress-induced activation. EMBO Rep. 9, 179–186 10.1038/sj.embor.7401157 (doi:10.1038/sj.embor.7401157) 18202720 PMC2246415

[20] Garrison SR Dietrich A Stucky CL 2011 TRPC1 contributes to light-touch sensation and mechanical responses in low-threshold cutaneous sensory neurons. J. Neurophysiol. 107, 913–922 10.1152/jn.00658.2011 (doi:10.1152/jn.00658.2011) 22072513 PMC3289471

[21] Huber TB 2006 Podocin and MEC-2 bind cholesterol to regulate the activity of associated ion channels. Proc. Natl Acad. Sci. USA 103, 17 079–17 086 10.1073/pnas.0607465103 (doi:10.1073/pnas.0607465103) PMC1859892 17079490

[22] Abrahamsen B Zhao J Asante CO Cendan CM Marsh S Martinez-Barbera JP Nassar MA Dickenson AH Wood JN 2008 The cell and molecular basis of mechanical, cold, and inflammatory pain. Science 321, 702–705 10.1126/science.1156916 (doi:10.1126/science.1156916) 18669863

[23] Elg S Marmigere F Mattsson JP Ernfors P 2007 Cellular subtype distribution and developmental regulation of TRPC channel members in the mouse dorsal root ganglion. J. Comp. Neurol. 503, 35–46 10.1002/cne.21351 (doi:10.1002/cne.21351) 17480026

[24] Goel M Sinkins WG Schilling WP 2002 Selective association of TRPC channel subunits in rat brain synaptosomes. J. Biol. Chem. 277, 48 303–48 310 10.1074/jbc.M207882200 (doi:10.1074/jbc.M207882200) 12377790

[25] Shin YC Shin SY So I Kwon D Jeon JH 2011 TRIP Database: a manually curated database of protein–protein interactions for mammalian TRP channels. Nucleic Acids Res. 39, D356–D361 10.1093/nar/gkq814 (doi:10.1093/nar/gkq814) 20851834 PMC3013757

[26] Drew LJ Rohrer DK Price MP Blaver KE Cockayne DA Cesare P Wood JN 2004 Acid-sensing ion channels ASIC2 and ASIC3 do not contribute to mechanically activated currents in mammalian sensory neurones. J. Physiol. 556, 691–710 10.1113/jphysiol.2003.058693 (doi:10.1113/jphysiol.2003.058693) 14990679 PMC1664992

[27] Hu J Lewin GR 2006 Mechanosensitive currents in the neurites of cultured mouse sensory neurones. J. Physiol. 577, 815–828 10.1113/jphysiol.2006.117648 (doi:10.1113/jphysiol.2006.117648) 17038434 PMC1804210

[28] Minasyan A 2009 Vestibular dysfunction in vitamin D receptor mutant mice. J. Steroid Biochem. Mol. Biol. 114, 161–166 10.1016/j.jsbmb.2009.01.020 (doi:10.1016/j.jsbmb.2009.01.020) 19429446

[29] Henry KR McGinn MD Carter LA Savoska EA 1992 Auditory brainstem function of the F1 offspring of the cross of CBA/CaJ and AU/SsJ inbred mice. Audiology 31, 190–195 10.3109/00206099209081654 (doi:10.3109/00206099209081654) 1444930

[30] Zheng QY Johnson KR Erway LC 1999 Assessment of hearing in 80 inbred strains of mice by ABR threshold analyses. Hear. Res. 130, 94–107 10.1016/S0378-5955(99)00003-9 (doi:10.1016/S0378-5955(99)00003-9) 10320101 PMC2855304

[31] Russell IJ Richardson GP 1987 The morphology and physiology of hair cells in organotypic cultures of the mouse cochlea. Hear. Res. 31, 9–24 10.1016/0378-5955(87)90210-3 (doi:10.1016/0378-5955(87)90210-3) 3429352

[32] Crawford AC Evans MG Fettiplace R 1989 Activation and adaptation of transducer currents in turtle hair cells. J. Physiol. 419, 405–434 2621635 10.1113/jphysiol.1989.sp017878 PMC1190013

[33] Kros CJ Rusch A Richardson GP 1992 Mechano-electrical transducer currents in hair cells of the cultured neonatal mouse cochlea. Proc. R. Soc. Lond. B 249, 185–193 10.1098/rspb.1992.0102 (doi:10.1098/rspb.1992.0102) 1280836

[34] Geleoc GS Lennan GW Richardson GP Kros CJ 1997 A quantitative comparison of mechanoelectrical transduction in vestibular and auditory hair cells of neonatal mice. Proc. R. Soc. Lond. B 264, 611–621 10.1098/rspb.1997.0087 (doi:10.1098/rspb.1997.0087) PMC1688386 9149428

[35] He DZ Jia S Dallos P 2004 Mechanoelectrical transduction of adult outer hair cells studied in a gerbil hemicochlea. Nature 429, 766–770 10.1038/nature02591 (doi:10.1038/nature02591) 15201911

[36] Johnson SL Beurg M Marcotti W Fettiplace R 2011 Prestin-driven cochlear amplification is not limited by the outer hair cell membrane time constant. Neuron 70, 1143–1154 10.1016/j.neuron.2011.04.024 (doi:10.1016/j.neuron.2011.04.024) 21689600 PMC3143834

[37] Beurg M Evans MG Hackney CM Fettiplace R 2006 A large-conductance calcium-selective mechanotransducer channel in mammalian cochlear hair cells. J. Neurosci. 26, 10 992–11 000 10.1523/JNEUROSCI.2188-06.2006 (doi:10.1523/JNEUROSCI.2188-06.2006) PMC6674673 17065441

[38] Spassova MA Hewavitharana T Xu W Soboloff J Gill DL 2006 A common mechanism underlies stretch activation and receptor activation of TRPC6 channels. Proc. Natl Acad. Sci. USA 103, 16 586–16 591 10.1073/pnas.0606894103 (doi:10.1073/pnas.0606894103) PMC1637625 17056714

[39] Gottlieb P 2008 Revisiting TRPC1 and TRPC6 mechanosensitivity. Pflugers Arch. Eur. J. Physiol. 455, 1097–1103 10.1007/s00424-007-0359-3 (doi:10.1007/s00424-007-0359-3) 17957383

[40] Wood JN 1990 Novel cell lines display properties of nociceptive sensory neurons. Proc. R. Soc. Lond. B 241, 187–194 10.1098/rspb.1990.0084 (doi:10.1098/rspb.1990.0084) 1979443

[41] Ostman JAR Nassar MA Wood JN Baker MD 2008 GTP up-regulated persistent Na^+^ current and enhanced nociceptor excitability require NaV1.9. J. Physiol. 586, 1077–1087 10.1113/jphysiol.2007.147942 (doi:10.1113/jphysiol.2007.147942) 18096591 PMC2268982

[42] Bae C Sachs F Gottlieb PA 2011 The mechanosensitive ion channel Piezo1 is inhibited by the peptide GsMTx4. Biochemistry 50, 6295–6300 10.1021/bi200770q (doi:10.1021/bi200770q) 21696149 PMC3169095

[43] Schnitzler M Storch U Meibers S Nurwakagari P Breit A Essin K Gollasch M Gudermann T 2008 Gq-coupled receptors as mechanosensors mediating myogenic vasoconstriction. EMBO J. 27, 3092–3103 10.1038/emboj.2008.233 (doi:10.1038/emboj.2008.233) 18987636 PMC2599876

[44] Lumpkin EA Caterina MJ 2007 Mechanisms of sensory transduction in the skin. Nature 445, 858–865 10.1038/nature05662 (doi:10.1038/nature05662) 17314972

[45] Drew LJ 2007 High-threshold mechanosensitive ion channels blocked by a novel conopeptide mediate pressure-evoked pain. PLoS ONE 2, e515 10.1371/journal.pone.0000515 (doi:10.1371/journal.pone.0000515) 17565368 PMC1885214

[46] Seal RP Wang X Guan Y Raja SN Woodbury CJ Basbaum AI Edwards RH 2009 Injury-induced mechanical hypersensitivity requires C-low threshold mechanoreceptors. Nature 462, 651–655 10.1038/nature08505 (doi:10.1038/nature08505) 19915548 PMC2810205

[47] Kroese AB Das A Hudspeth AJ 1989 Blockage of the transduction channels of hair cells in the bullfrog's sacculus by aminoglycoside antibiotics. Hear. Res. 37, 203–217 10.1016/0378-5955(89)90023-3 (doi:10.1016/0378-5955(89)90023-3) 2468634

[48] Rusch A Kros CJ Richardson GP 1994 Block by amiloride and its derivatives of mechano-electrical transduction in outer hair cells of mouse cochlear cultures. J. Physiol. 474, 75–86 7516972 10.1113/jphysiol.1994.sp020004 PMC1160297

[49] Farris HE LeBlanc CL Goswami J Ricci AJ 2004 Probing the pore of the auditory hair cell mechanotransducer channel in turtle. J. Physiol. 558, 769–792 10.1113/jphysiol.2004.061267 (doi:10.1113/jphysiol.2004.061267) 15181168 PMC1665030

[50] Marcotti W Geleoc GS Lennan GW Kros CJ 1999 Transient expression of an inwardly rectifying potassium conductance in developing inner and outer hair cells along the mouse cochlea. Pflugers Arch. 439, 113–122 10.1007/s004240051134 (doi:10.1007/s004240051134) 10651007

[51] Gale JE Marcotti W Kennedy HJ Kros CJ Richardson GP 2001 FM1-43 dye behaves as a permeant blocker of the hair-cell mechanotransducer channel. J. Neurosci. 21, 7013–7025 11549711 10.1523/JNEUROSCI.21-18-07013.2001 PMC6762973

[52] Drew LJ Wood JN 2007 FM1-43 is a permeant blocker of mechanosensitive ion channels in sensory neurons and inhibits behavioural responses to mechanical stimuli. Mol. Pain 3, 1 10.1186/1744-8069-3-1 (doi:10.1186/1744-8069-3-1) PMC1779769 17207285

[53] Shen J Harada N Kubo N Liu B Mizuno A Suzuki M Yamashita T 2006 Functional expression of transient receptor potential vanilloid 4 in the mouse cochlea. Neuroreport 17, 135–139 10.1097/01.wnr.0000199459.16789.75 (doi:10.1097/01.wnr.0000199459.16789.75) 16407759

[54] Cable J Steel KP 1998 Combined cochleo-saccular and neuroepithelial abnormalities in the Varitint-waddler-J (VaJ) mouse. Hear. Res. 123, 125–136 10.1016/S0378-5955(98)00107-5 (doi:10.1016/S0378-5955(98)00107-5) 9745961

[55] Di PF Belyantseva IA Kim HJ Vogt TF Kachar B Noben-Trauth K. 2002 Mutations in Mcoln3 associated with deafness and pigmentation defects in varitint-waddler (Va) mice. Proc. Natl Acad. Sci. USA 99, 14 994–14 999 10.1073/pnas.222425399 (doi:10.1073/pnas.222425399) PMC137533 12403827

[56] Grimm C Cuajungco MP van Aken AFJ Schnee M Jors S Kros CJ Ricci AJ Heller S 2007 A helix-breaking mutation in TRPML3 leads to constitutive activity underlying deafness in the varitint-waddler mouse. Proc. Natl Acad. Sci. USA 104, 19 583–19 588 10.1073/pnas.0709846104 (doi:10.1073/pnas.0709846104) PMC2148332 18048323

[57] van Aken AF Atiba-Davies M Marcotti W Goodyear RJ Bryant JE Richardson GP Noben-Trauth K Kros CJ 2008 TRPML3 mutations cause impaired mechano-electrical transduction and depolarization by an inward-rectifier cation current in auditory hair cells of varitint-waddler mice. J. Physiol. 586, 5403–5418 10.1113/jphysiol.2008.156992 (doi:10.1113/jphysiol.2008.156992) 18801844 PMC2655368

[58] Bautista DM Jordt S-E Nikai T Tsuruda PR Read AJ Poblete J Yamoah EN Basbaum AI Julius D 2006 TRPA1 mediates the inflammatory actions of environmental irritants and proalgesic agents. Cell 124, 1269–1282 10.1016/j.cell.2006.02.023 (doi:10.1016/j.cell.2006.02.023) 16564016

[59] Takumida M Anniko M 2009 Expression of canonical transient receptor potential channel (TRPC) 1–7 in the mouse inner ear. Acta Otolaryngol. 129, 1351–1358 10.3109/00016480902798350 (doi:10.3109/00016480902798350) 19922081

[60] Amiri H Schultz G Schaefer M 2003 FRET-based analysis of TRPC subunit stoichiometry. Cell Calcium 33, 463–470 10.1016/S0143-4160(03)00061-7 (doi:10.1016/S0143-4160(03)00061-7) 12765691

[61] Kros CJ Crawford AC 1990 Potassium currents in inner hair cells isolated from the guinea-pig cochlea. J. Physiol. 421, 263–291 2348394 10.1113/jphysiol.1990.sp017944 PMC1190084

[62] Kros CJ Ruppersberg JP Rusch A 1998 Expression of a potassium current in inner hair cells during development of hearing in mice. Nature 394, 281–284 10.1038/28401 (doi:10.1038/28401) 9685158

[63] Pyott SJ Meredith AL Fodor AA Vazquez AE Yamoah EN Aldrich RW 2007 Cochlear function in mice lacking the BK channel alpha, beta1, or beta4 subunits. J. Biol. Chem. 282, 3312–3324 10.1074/jbc.M608726200 (doi:10.1074/jbc.M608726200) 17135251

[64] Dietrich A 2005 Increased vascular smooth muscle contractility in TRPC6–/– mice. Mol. Cell. Biol. 25, 6980–6989 [Erratum in Mol. Cell Biol. 2005 25, 11 191.] (doi:10.1128/MCB.25.16.6980-6989.2005) 16055711 10.1128/MCB.25.16.6980-6989.2005 PMC1190236

[65] Okuse K Malik-Hall M Baker MD Poon W-YL Kong H Chao MV Wood JN 2002 Annexin II light chain regulates sensory neuron-specific sodium channel expression. Nature 417, 653–656 10.1038/nature00781 (doi:10.1038/nature00781) 12050667

[66] Raybould NP Jagger DJ Kanjhan R Greenwood D Laslo P Hoya N Soeller C Cannell MB Housley GD 2007 TRPC-like conductance mediates restoration of intracellular Ca^2+^ in cochlear outer hair cells in the guinea pig and rat. J. Physiol. 579, 101–113 10.1113/jphysiol.2006.122929 (doi:10.1113/jphysiol.2006.122929) 17158171 PMC2075380

[67] Chaplan SR Bach FW Pogrel JW Chung JM Yaksh TL 1994 Quantitative assessment of tactile allodynia in the rat paw. J. Neurosci. Methods 53, 55–63 10.1016/0165-0270(94)90144-9 (doi:10.1016/0165-0270(94)90144-9) 7990513

[68] Pau H Hawker K Fuchs H de Angelis MH Steel KP 2004 Characterization of a new mouse mutant, flouncer, with a balance defect and inner ear malformation. Otol. Neurotol. 25, 707–713 10.1097/00129492-200409000-00010 (doi:10.1097/00129492-200409000-00010) 15353999

[69] Kiernan AE Zalzman M Fuchs H de Angelis MH Balling R Steel KP Avraham KB 1999 Tailchaser (Tlc): a new mouse mutation affecting hair bundle differentiation and hair cell survival. J. Neurocytol. 28, 969–985 10.1023/A:1007090626294 (doi:10.1023/A:1007090626294) 10900098

[70] Cox 2006 An SCN9A channelopathy causes congenital inability to experience pain. Nature 444, 894–898 10.1038/nature05413 (doi:10.1038/nature05413) 17167479 PMC7212082

