# Supplementary material for: TRPC3 and TRPC6 are essential for normal mechanotransduction in subsets of sensory neurons and cochlear hair cells
Source: Open Biol. 2012 May;2(5):120068. doi: 10.1098/rsob.120068 (PMC3376737; doi:10.1098/rsob.120068)
Supplement: Electronic supplementary material [file rsob120068-s1.doc]

**Supplemental Data**

**Figure S1, related to Figure 1 – Distribution of Peripherin, NF200, IB4 and CGRP in the DRG and spinal cord**


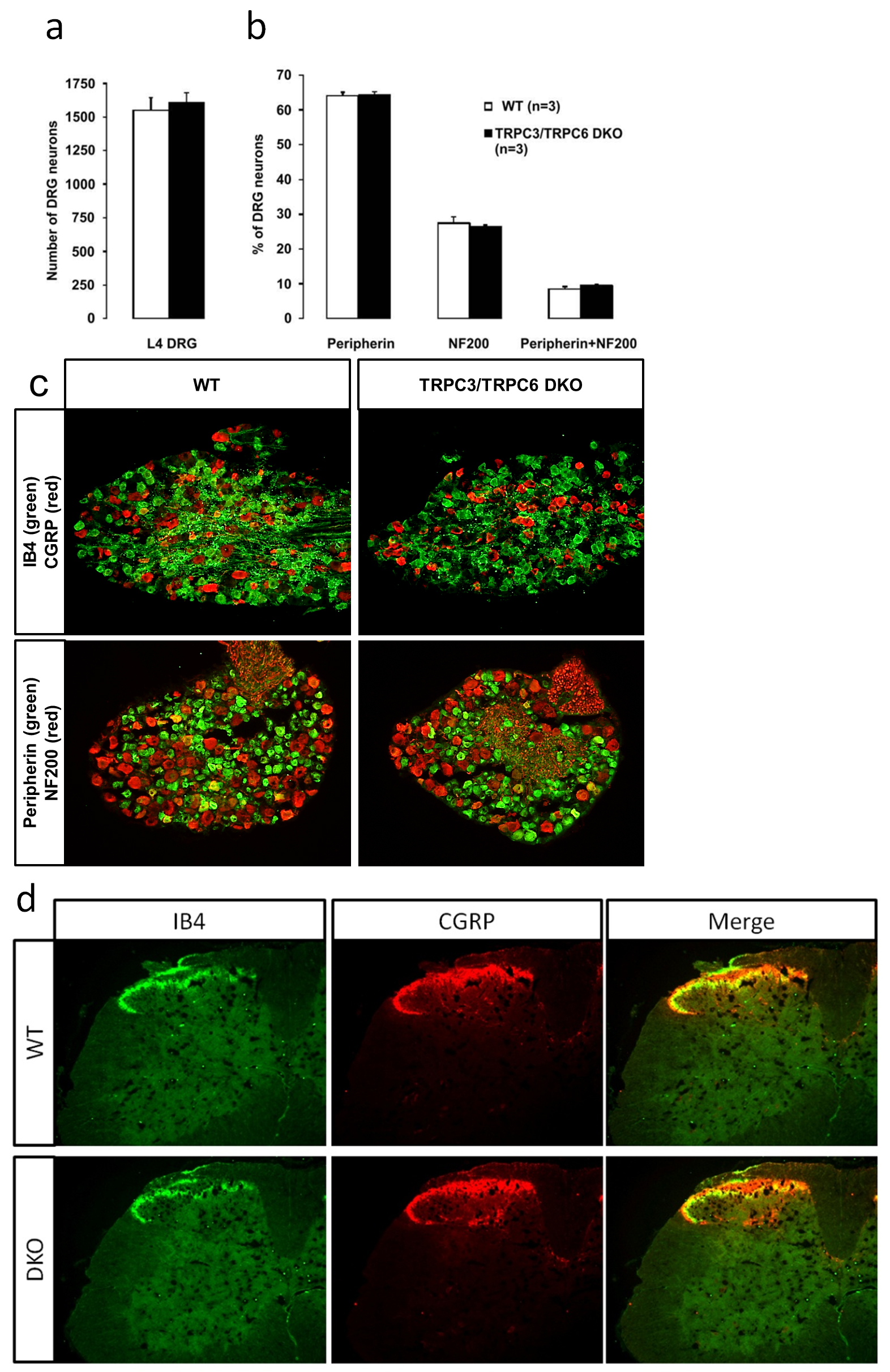


**(a)** Total number of DRG neurons, as calculated by the sum of Peripherin positive, Neurofilament 200 (NF200) positive and double-positive neurons. **(b)** Percentage of DRG neurons stained by only Peripherin, NF200 or Peripherin and NF200. Data in a/b is expressed as mean±SEM. **(c)** Exemplar staining of the subpopulation markers of sensory neurons: Isolectin B4 (IB4), CGRP, Peripherin and NF200 in WT and TRPC3/TRPC6 DKO DRG **(d)** Cross sections of Lumbar 4-5 from spinal cord were labelled with IB4 (left panel) and anti-CGRP (middle panel). The right panels are merged images of the left and middle panels. Identical expression patterns of IB4 positive terminals (green, lamina I and II inner) and CGRP positive terminals (red, lamina I and II outer) were detected in the lumbar superficial dorsal horn from both WT (top) and TRPC3/TRPC6 DKO (bottom) mice.

**Figure S2, related to Figure 2 – Motor coordination on rotarod**


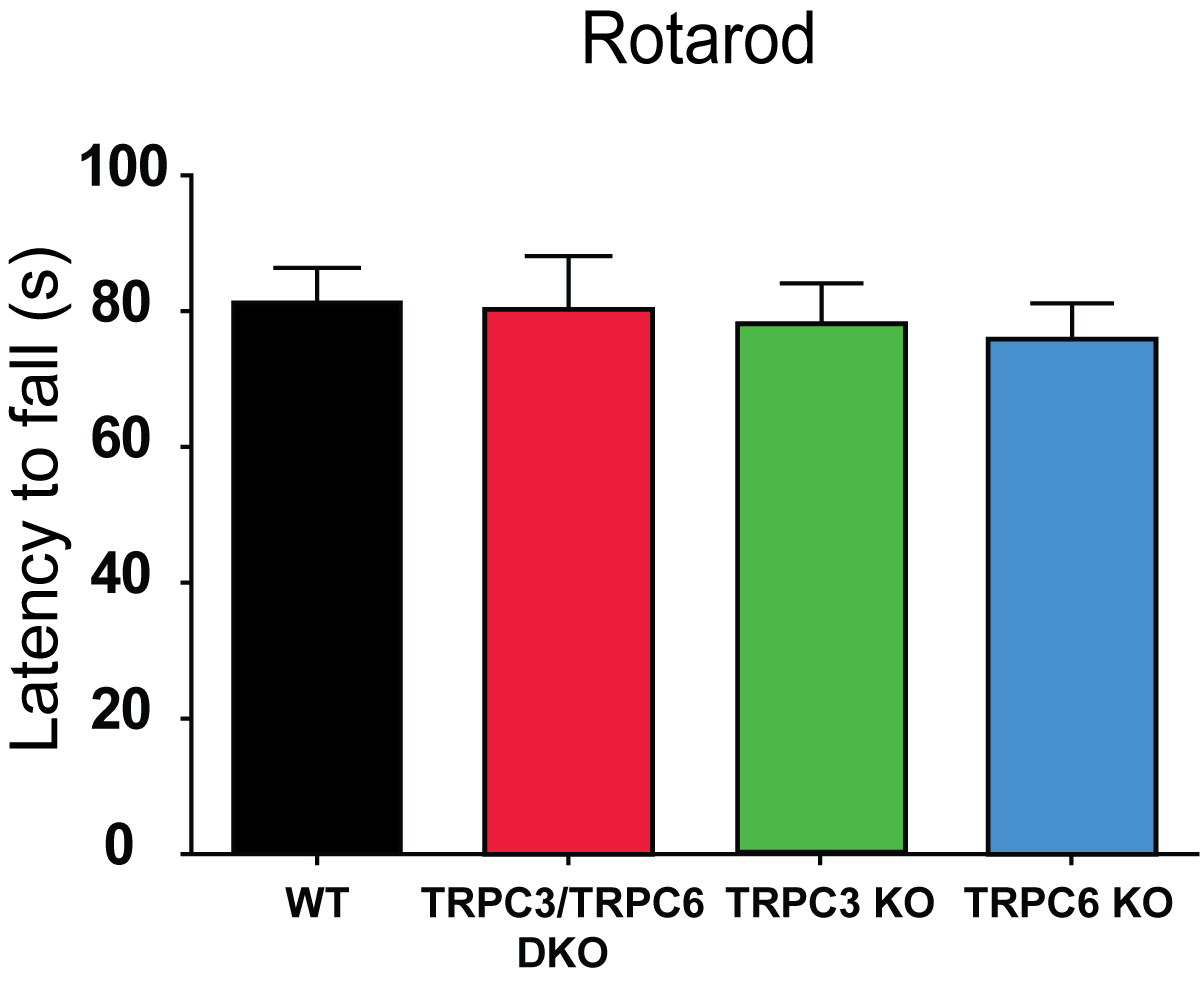


Performance of wild type (WT), TRPC3/TRPC6 double knock-out (DKO), TRPC3 knock-out (KO) and TRPC6 KO mice on a rotarod (n=6/group). Data are expressed as mean±SEM.

**Figure S3, related to Figure 5 – RT-PCR of TRPC3 and TRPC6 in cultured hair cells**


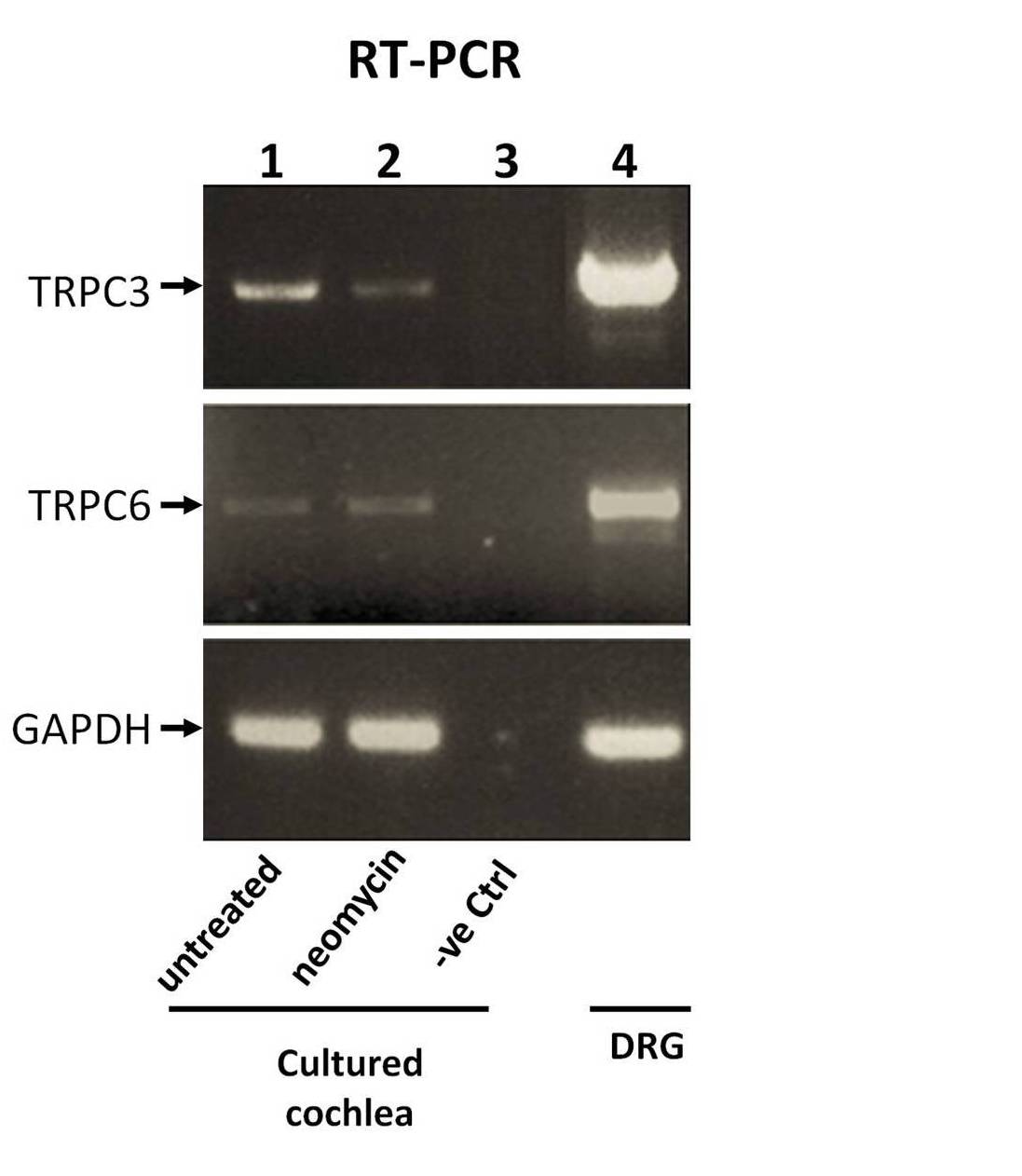


TRPC3 and TRPC6 transcripts from DRG and cultured cochlea were detected with reverse transcription-PCR (RT-PCR). The hair cells in cochlea were killed by neomycin treatment during culture. TRPC3 and TRPC6 bands were amplified from untreated cochlea (lane 1), neomycin treated cochlea (lane 2) and DRG (lane 4) by PCR. The same amount of total RNA from cultured cochlea was used as PCR template for negative control (lane 3). GAPDH primers were used as an internal loading control.

**Figure S4, related to Figure 8 – Whole cell current responses to mechanical stimulation**


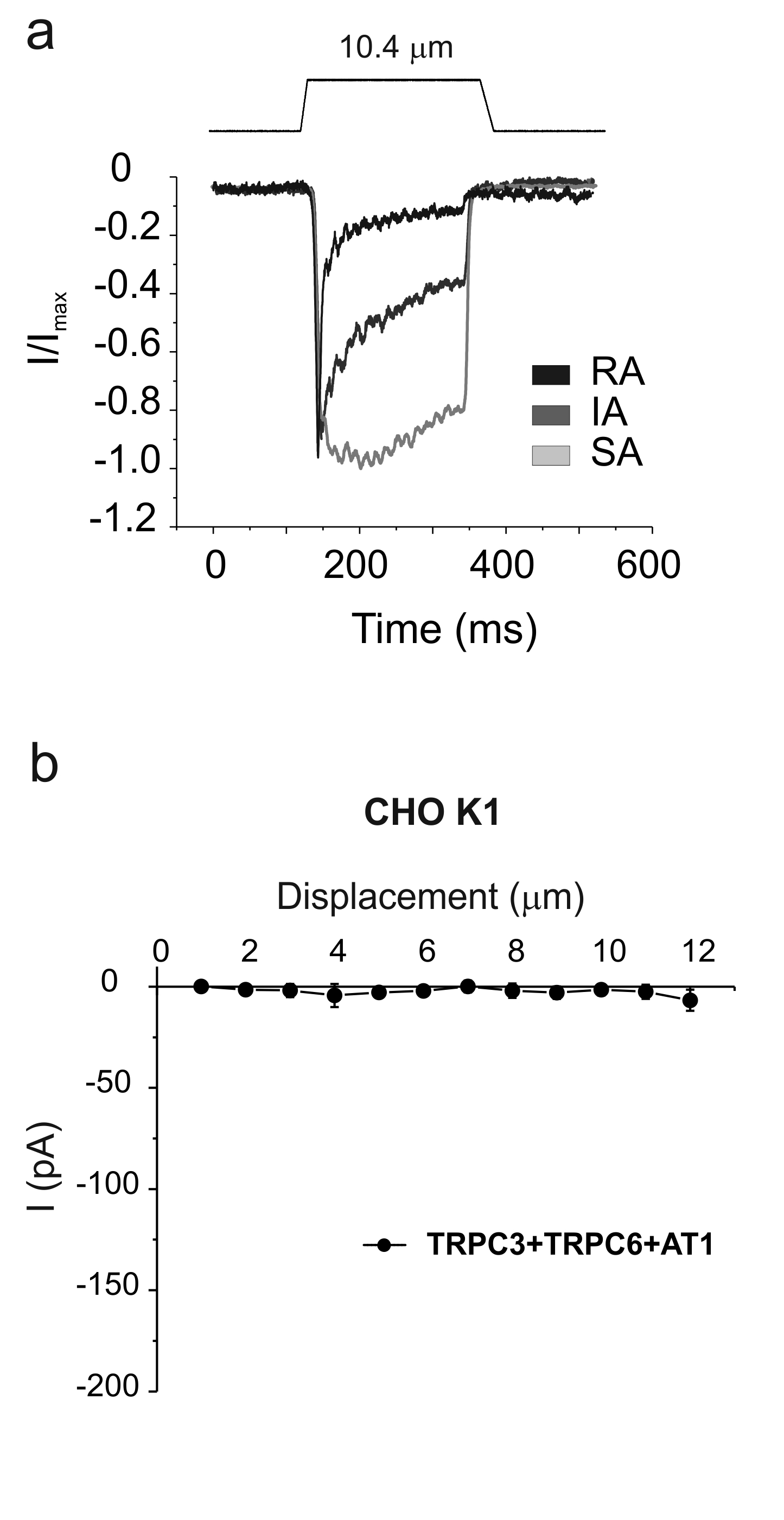


(a) Mean whole cell voltage clamp recordings of mechanically activated currents from small diameter DRG neurons. Rapidly adapting (RA), intermediately adapting (IA) and slowly adapting (SA). Holding potential was -70 mV. (b) Mean mechanically evoked inward currents from CHO cells overexpressing hTRPC3, hTRPC6 and the angiotensin II receptor (AT1). Data are expressed as mean±SEM.

**Figure S5, related to Figure 1 - Generation of TRPC3/TRPC6 double knock-out mice**


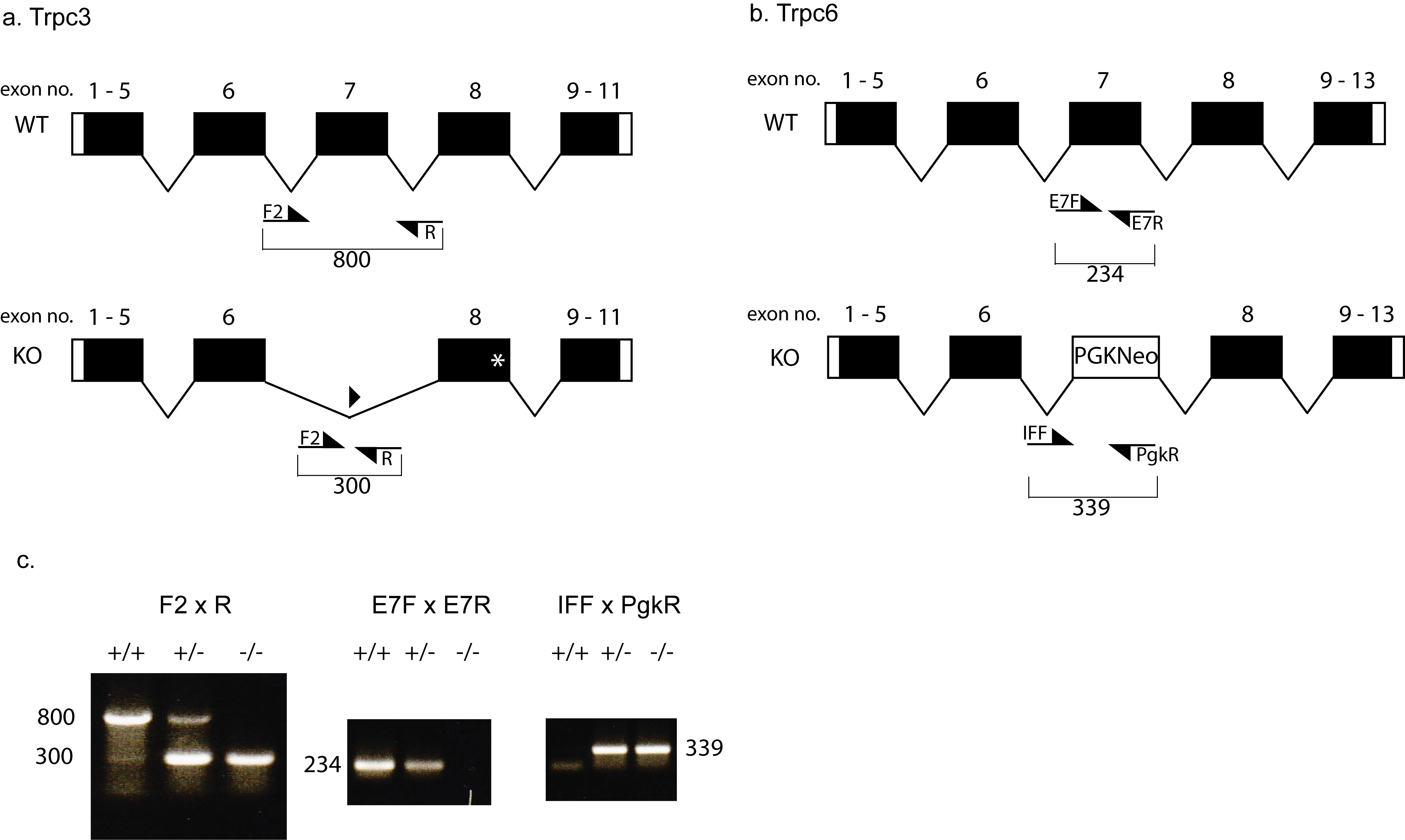


**(a)** (Top) Diagram of the intron-exon organisation of the *Mus musculus* TRPC3 gene. The lengths of the amplicons, including primers, are depicted. PCR primers; F2 and R, stop codon; *, loxP; black triangle. (Bottom) Diagram of the expected disruption after excision of exon 7 by the action of Cre recombinase. **(b)** (Top) Diagram of the intron-exon organisation of the Mus musculus TRPC6 gene. The lengths of the amplicons, including primers, are depicted. PCR primers; E7F, E7R, IFF and PgkR. (Bottom) Diagram of the TRPC6 gene after targeted disruption by the PGKNeo cassette. **(c)** Image of the electrophoretic migration in an agarose gel of the amplicons obtained using the indicated primers

**Supplementary movies S1 – S4 Preyer reflex response, related to Figure 5**

Mice were acclimatized to the environment for 1 hour and their response to a click box stimulus (90dsb, 20 kHz) was videotaped. The responses were scored blind as described in the methods, an example recording from each genotype is shown in the supplementary material.

(Movie S1) Wild type mouse displays a Preyer reflex with both ears going flat against the head in response to the click box. TRPC3 knock-out mice (Movie S2) and TRPC6 knock-out mice (Movie S3) also display a strong Preyer reflex. (Movie S4) TRPC3/TRPC6 double knock-out mice do not respond to the click box stimulus with a Preyer reflex, both ears remain static.

**Supplementary movie S5 Swim test, related to Figure 5**

Mice were placed into deep water for 1 minute and swimming behaviour whilst swimming was observed. The video shows examples of swimming behaviour from wild type and TRPC3/TRPC6 double knock-out mice. Balance relies on the vestibular system together with visual and proprioceptive inputs. When in water the proprioceptive inputs are removed and the vestibular system is crucial for balance and to stay above the water. The wild type mice swim in a horizontal position throughout the swim test and the head is above water. In contrast the TRPC3/TRPC6 double knock-out mice swim in a vertical position for the majority of the swim test and the head submerges below the water on many occasions indicating partial vestibular system dysfunction.

**Supplemental Experimental Procedures**

**Immunohistochemistry**

To prepare the DRG sections for lectin IB4 (IB4) and calcitonin gene-related peptide (CGRP) staining, mice were deeply anesthetised with pentobarbital (140 mg/kg) and transcardially perfused with heparinised saline (0.9% NaCl) followed by 4% paraformaldehyde (PFA) in 0.1 M phosphate buffer (PB), pH 7.4. The lumbar DRGs and spinal cord (from lumbar level 2 to lumbar 5) were dissected and post-fixed in 4% PFA overnight at 4°C. The tissue was transferred into 20% sucrose in 0.1 M PB for 24 hours at 4°C and then embedded in O.C.T. compound (BDH Essex, UK) on dry ice. Cryostat sections (thickness, 11μm for DRG and 20 μm for spinal cord) were cut and collected on Superfrost plus slides. Preparation for peripherin and neurofilament 200 (NF200) staining were prepared as follows. Fresh unperfused DRGs from lumbar level L4 of wild type control and TRPC3/TRPC6 double knock-out mice were dissected and embedded in O.C.T. compound. Cryostat sections (thickness, 11μm) were serially cut. Every 8th section throughout the L4 DRG was collected. All the slides were dried over night at room temperature after sectioning. The sections for Peripherin and NF200 staining were fixed with fresh prepared 4% PFA at room temperature for 7 minutes, and then washed three times for 5 minutes each with 1x PBS containing 0.3% Triton X-100 (PBST). All the sections for immunostaining were incubated in blocking buffer (PBST containing 10% goat serum, Sigma G9023) at room temperature for 1 hour and then were incubated with diluted primary antibodies overnight at 4°C. After three times washing for 10 minutes with PBST, sections were incubated with diluted secondary antibodies at room temperature for 2 hours. The slides were washed with PBST for three times 10 minutes each and then mounted using VECTASHIELD HardSet Mounting Medium (Vector Labs, H-1400). Images were captured using a Hamamatsu Camera attached to a microscope (Leica) using HC Image Live software Exposure times of photographs were identical for all slices. All stainings were done in parallel for spinal and DRG of wild type and TRPC3 and TRPC6 double knock-out mice. The number of DRG neurons was counted as described 1. Briefly, the number of peripherin-positive (green), NF200-positive (red) and double-positive (yellow) neurons were counted blind in all sections from L4 DRG in each animal. The total number of L4 DRG neurons was calculated by adding Peripherin-, NF200- and double-positive neuron numbers together. The following primary antibodies were diluted in blocking buffer: rabbit anti-CGRP (1:4000, Sigma C8198); mouse anti-Peripherin (1:500, Sigma, P5117); rabbit anti-Neurofilament 200 (1:200, Sigma, UK, N4142). Lectin IB4-biotin (1:500, Sigma, L2140) was diluted in PBS containing 0.1 mM MnCl2, 0.1 mM MgCl2, and 0.1 mM CaCl2. The following secondary antibodies were diluted in blocking buffer: goat anti-mouse IgG Alexa fluor 488 (1:1000, Invitrogen, A11017); goat anti-rabbit IgG Alexa fluor 594 (1:1000, Invitrogen, A11037) and Alexa Fluor 488-conjugated streptavidin (1:200, Invitrogen, S11223).

**Cochlear cultures and PCR sample preparation**

DRG were dissected from adult C57BL/6 mice and stored in RNAlater for RNA isolation. Cochlear cultures were established from 2 day postnatal CD1 mouse pups on collagen coated glass coverslips in Maximows slide assemblies using a medium containing 93% DMEM/F12, 7% foetal bovine serum and 10 µg/ml ampillicin. After 24 hours the coverslips with the adherent cochlear cultures were transferred to 35 mm diameter plastic petri dishes and fed with 2.5 ml of the same medium. To kill hair cells neomycin sulphate was added to a final concentration of 200 μM for the first 24 hours, and then to 400 μM for the following 24 hours. Control cultures were treated with a volume of saline equivalent to that of the neomycin stock solution added. After the 48 hour treatment, cultures were washed 3 times with 2 ml of sterile saline, removed from the coverslips and placed in RNAlater.

**RNA isolation and reverse transcription-PCR (RT-PCR)**

Total RNA was extracted either from cultured cochlea or DRG using Trizol reagent (Invitrogen) and Phase Lock Gel Heavy (5 PRIME). RNA samples were treated with DNase and purified with RNeasy MinElute Cleanup kit (Qiagen). cDNA reverse transcription from the RNA sample was performed using iScript cDNA Synthesis Kit (Bio-rad) according to the manufacturer’s instructions. Five hundred nanogram of total RNA was subjected to in vitro reverse transcription. PCR was performed using Phusion High-Fidelity PCR Kit (New England Biolabs) with specific TRPC3 and TRPC6 primers (see below). The initial denaturation step was 98°C for 30 seconds, and reactions were amplified for 35 cycles at 98°C for 8 seconds, 66.6°C/64°C (TRPC3/TRPC6) for 20 seconds, and 72°C for 1.5 minutes, followed by final incubation at 72°C for 10 minutes. PCR products were separated in 0.8% agarose gels. The sizes of TRPC3 and TRPC6 PCR products are 2.8 kb (TRPC3) and 2.5 kb (TRPC6), respectively. GAPDH primers were used as an internal control. The size of the amplicon of GAPDH is 200 bp.

TRPC3 forward, 5’- AGCTGGGCTGCTGACTGCG -3’

TRPC3 reverse, 5’- CGTTGTGCTATAGTCAAGGCTTG -3’

TRPC6 forward, 5’- CCAGCTTCCGGGGTAATG -3’

TRPC6 reverse, 5’- TCCTTCAATTCCCCTTCATTC -3’

GAPDH forward, 5’- TGCGACTTCAACAGCAACTC -3’

GAPDH reverse, 5’- CTTGCTCAGTGTCCTTGCTG -3’

| **Current findings** | **Phenotype after deletion** | |
| --- | --- | --- |
|  | ***DRG*** | ***Cochlea*** |
| TRPC3/TRPC6 | **Loss of mechanically-evoked RA currents**  **Increased threshold to light touch** | **Loss of MA currents in OHC from basal end**  **Loss of high frequency hearing** |
| TRPC3 | **Switch of mechanically-evoked currents from a RA to IA** | **/** |
| TRPC6 | **/** | **/** |
| **Other TRP findings** | **Phenotype of other TRP channel deletion** | |
|  | ***DRG*** | ***Cochlea*** |
| TRPC1 | Decrease in mechanically-evoked AP  Increased threshold to light touch (Garrison et al., 2011) | ? |
| TRPA1 | Loss of mechanically-evoked SA current  Increased threshold to noxious mechanically stimuli(Kwan et al., 2009) | Enhanced sensitivity to high frequency hearing(Kwan et al., 2006) |
| TRPV4 | Contribution to mechanically-evoked visceral pain and inflammatory hyperalgesia (Alessandri-Haber et al., 2009) | Age-related hearing loss (Cuajungco et al., 2007) |

**Supplementary Table 1: Potential role of TRPs in mechanotransduction**

Overexpression TRPC3 conferred high threshold mechanosensitivity in a sensory cell line, while overexpression of TRPC3 andTRPC6 conferred low threshold mechnosensitivity in a sensory neuron cell line. No effect was observed in non-neuronal cell lines. Currently no other evidence is present that overexpression of particular TRPs confer mechanosensitivity to cell lines

DRG: dorsal root ganglia; MA: mechanically-activated; OHC: outer hair cell; RA: rapidly-adapting; IA: intermediately-adapting; SA: slowly-adapting; AP: action potential.

Reference List

Alessandri-Haber,N., Dina,O.A., Chen,X., and Levine,J.D. (2009). TRPC1 and TRPC6 channels cooperate with TRPV4 to mediate mechanical hyperalgesia and nociceptor sensitization. J. Neurosci. *29*, 6217-6228.

Cuajungco,M.P., Grimm,C., and Heller,S. (2007). TRP channels as candidates for hearing and balance abnormalities in vertebrates. Biochim. Biophys. Acta *1772*, 1022-1027.

Garrison,S.R., Dietrich,A., and Stucky,C.L. (2011). TRPC1 contributes to light-touch sensation and mechanical responses in low-threshold cutaneous sensory neurons. J Neurophysiol.

Kwan,K.Y., Glazer,J.M., Corey,D.P., Rice,F.L., and Stucky,C.L. (2009). TRPA1 modulates mechanotransduction in cutaneous sensory neurons. J Neurosci. *29*, 4808-4819.

Kwan,K.Y., Allchorne,A.J., Vollrath,M.A., Christensen,A.P., Zhang,D.S., Woolf,C.J., and Corey,D.P. (2006). TRPA1 Contributes to Cold, Mechanical, and Chemical Nociception but Is Not Essential for Hair-Cell Transduction. Neuron *50*, 277-289.
